# Combining metabolic flux analysis with proteomics to shed light on the metabolic flexibility: the case of *Desulfovibrio vulgaris* Hildenborough

**DOI:** 10.3389/fmicb.2024.1336360

**Published:** 2024-02-23

**Authors:** Xavier Marbehan, Magali Roger, Frantz Fournier, Pascale Infossi, Emmanuel Guedon, Louis Delecourt, Régine Lebrun, Marie-Thérèse Giudici-Orticoni, Stéphane Delaunay

**Affiliations:** ^1^LRGP, Université de Lorraine, CNRS, Nancy, France; ^2^BIP-UMR 7281, Laboratoire de Bioénergétique et Ingénierie des Protéines, Aix-Marseille Université, CNRS, Marseille, France; ^3^LISM-UMR 7255, Laboratoire d’Ingénierie des Systèmes Macromoléculaires, Aix-Marseille Université, CNRS, Marseille, France; ^4^IMM-FR3479, Marseille Protéomique, Aix-Marseille Université, CNRS, Marseille, France

**Keywords:** metabolic model, flux balance analysis, proteomic, *Desulfovibrio vulgaris* Hildenborough, sulfate respiration, hydrogen metabolism, formate metabolism, metabolic flexibility

## Abstract

**Introduction:**

*Desulfovibrio vulgaris* Hildenborough is a gram-negative anaerobic bacterium belonging to the sulfate-reducing bacteria that exhibits highly versatile metabolism. By switching from one energy mode to another depending on nutrients availability in the environments„ it plays a central role in shaping ecosystems. Despite intensive efforts to study *D. vulgaris* energy metabolism at the genomic, biochemical and ecological level, bioenergetics in this microorganism remain far from being fully understood. Alternatively, metabolic modeling is a powerful tool to understand bioenergetics. However, all the current models for *D. vulgaris* appeared to be not easily adaptable to various environmental conditions.

**Methods:**

To lift off these limitations, here we constructed a novel transparent and robust metabolic model to explain *D. vulgaris* bioenergetics by combining whole-cell proteomic analysis with modeling approaches (Flux Balance Analysis).

**Results:**

The iDvu71 model showed over 0.95 correlation with experimental data. Further simulations allowed a detailed description of *D. vulgaris* metabolism in various conditions of growth. Altogether, the simulations run in this study highlighted the sulfate-to-lactate consumption ratio as a pivotal factor in *D. vulgaris* energy metabolism.

**Discussion:**

In particular, the impact on the hydrogen/formate balance and biomass synthesis is discussed. Overall, this study provides a novel insight into *D. vulgaris* metabolic flexibility.

## 1 Introduction

Sulfate reducing microorganisms (SRMs) are anaerobic bacteria and archaea, not phylogenetically related, coupling dissimilatory sulfate (SO_4_^2–^) reduction into sulfide (H_2_S) to the oxidation of organic compounds (such as lactate, pyruvate, formate or ethanol) or molecular H_2_ to support growth (Muyzer and Stams, 2008). SRMs are ubiquitous in anoxic environments such as flooded soils, sediments, landfills, lagoons, where they have an important role in sulfur and carbon cycles (Nriagu, 1985; Muyzer and Stams, 2008). In the absence of sulfate, they can also ferment a variety of organic substrates (such as lactate, pyruvate or ethanol) to acetate, hydrogen (H_2_) and carbon dioxide (CO_2_). This pathway is regulated by the H_2_-partial pressure and occurs mostly when H_2_-oxidizing microorganisms are present in the environment (such as methanogens) (Bryant et al., 1977; McInerney et al., 1979; Stolyar et al., 2007). Hence, depending on sulfate availability in the environment, SRMs can either contribute as intermediate in microbial degradation of complex organic matter in anoxic environments in syntrophy with methanogens, or compete with methanogens for hydrogen and acetate while providing electron donor to a great diversity of aerobic chemotrophic or anoxygenic phototrophic microorganisms (Muyzer and Stams, 2008). Therefore, SRMs play a central role in natural and engineered ecosystems (Wang et al., 2023). Besides, SRMs exhibit a great metabolic versatility, switching from one energy mode to another depending on nutrients availability, which allow them to adapt to various environmental conditions and persist into several ecological niches (Muyzer and Stams, 2008). SRMs, throughout the production of toxic and corrosive hydrogen sulfide, can cause significant damages to ecosystems (Malone Rubright et al., 2017). Beyond the environmental impact, they are also responsible of unwanted biocorrosion of ferrous metals (Enning and Garrelfs, 2014). Moreover, it is now well recognized that SRMs also contribute to human health disorders such as in Inflammatory Bowel diseases (Carbonero et al., 2012). Nevertheless, SRMs can also be exploited beneficially in industrial processes such as bio-capture and recovery of heavy metals from wastewater (Hao et al., 2014), or in depollution processes of toxic metals and radionuclides (Qian et al., 2019). Given the importance of SRMs in human health, environment and industrial microbiology, a fundamental understanding of SRMs energy metabolism is crucial for better prediction of SRMs activities in the environment. This will contribute to a better control of the entire ecosystem.

Among SRMs, *Desulfovibrio vulgaris* Hildenborough (*Dv*H), a Gram-negative anaerobic bacterium, is one representative of SRMs that has been extensively studied at the genomic, biochemical and ecological level (Heidelberg et al., 2004). While lactate is preferentially used, this bacterium can also utilize formate or ethanol as carbon and energy source (Heidelberg et al., 2004; Tao et al., 2007). In a sulfate-containing medium, the bacterium will perform sulfate respiration which leads to hydrogen sulfide synthesis. It was initially proposed that electrons and protons generated through lactate oxidation could serve as substrates to membrane-associated hydrogenases (Keller and Wall, 2011). The hydrogen produced could then diffuse across the inner membrane and be oxidized by periplasmic hydrogenases, thus providing electrons for sulfate reduction while the protons released from this reaction would contribute to chemiosmotic gradient (Keller and Wall, 2011). This energy conserving model called hydrogen-cycling is reinforced by the transient presence of hydrogen during *Dv*H growth (Smith et al., 2019). Alternatively, the oxidation of lactate could lead to the formation of formate through the pyruvate formate lyase (PFL) that catalyzes the interconversion of pyruvate into formate and acetyl-coA (Kazakov et al., 2015). In analogy with the hydrogen-cycling model, electrons and protons released from formate oxidation by periplasmic formate dehydrogenases (FDHs) can cycle back to the cytoplasm through transmembrane electron transport complexes (TMCs) for sulfate reduction while contributing to proton gradient across the membrane (Heidelberg et al., 2004). Using ATP synthase activity, this anaerobic respiration allows production of a higher amount of energy than fermentation. In comparison, fermentation alone generates 1 mol ATP per mol of lactate, whereas sulfate respiration generates 2.5 mol ATP per mol of lactate, showcasing its higher energy yield. Nevertheless, the concept of hydrogen- or formate-cycling remains controversial (Voordouw, 2002; Keller and Wall, 2011), and another model was proposed in which multiple electron transfer routes operate simultaneously: the hydrogen-/formate-cycling model and an independent pathway involving membrane-associated electron transfer complexes (Noguera et al., 1998; Sim et al., 2013).

To better understand the mechanisms involved in hydrogen and formate metabolisms, which are key metabolites in interspecies transfer within ecosystems, metabolic models could serve as powerful tools (Heirendt et al., 2019). Several metabolic models have been previously developed for *Dv*H. One model was reconstructed based on balance equations to study the carbon metabolite exchanges between *Dv*H and *Methanococcus maripaludis*, as well as their impact on the latter growth (Stolyar et al., 2007). Another model was developed to create and validate a numerical tool (OptCom) dedicated to the simulation of the complexity of metabolic exchanges within the context of a microbial consortium (Zomorrodi and Maranas, 2012). A third model investigated *Dv*H ability to adapt to its environment, particularly with regard to available energy sources, by implementing specific reactions such as those of the Wood–Ljungdahl pathway (Flowers et al., 2018). This genome-based model is, to date, the most comprehensive and complex metabolic model. More recently, a mass transfer model providing a macroscopic representation of SRM metabolism was also constructed (Smith et al., 2019). However, this model does not give any information on the metabolic fluxes of the respective pathways involved in SRM metabolism. To our knowledge, no models accurately captured yet the hydrogen-/formate metabolism in *Dv*H. Indeed, none of these models were designed to simulate production/utilization of hydrogen and formate by *Dv*H. Therefore, they are uncomplete in terms of pathways involved. Furthermore, they also suffer from limited available and consistent experimental dataset. The objective of the present study was to reconstruct a transparent and robust metabolic model for *Dv*H, combining proteomic-scale reconstruction, and metabolic analysis with the aim to investigate the hydrogen and formate metabolism of *Dv*H.

## 2 Materials and method

### 2.1 Media and growth conditions

Pure cultures of *Dv*H were typically grown at 37°C under anaerobic conditions to mid-exponential phase in Hungate tubes in Starkey (SKY) medium containing 0.5 g L^–1^ K_2_HPO_4_; 2 g L^–1^ NH_4_Cl; 2 g L^–1^ MgSO_4_, 7⋅H_2_O; 4 g L^–1^ Na_2_SO_4_; 1 g L^–1^ yeast extract (Difco laboratories), 3 6 g L^–1^ sodium lactate (adapted from Starkey, 1948); 1 mL L^–1^ trace elements [10.75 g L^–1^ MgO; 2 g L^–1^ CaCO_3_; 6.2 g L^–1^ FeSO_4_, 7⋅H_2_O; 1.44 g L^–1^ ZnSO_4_, 7⋅H_2_O; 0.84 g L^–1^ MnSO_4_, 7⋅H_2_O; 0.25 g L^–1^ CuSO_4_, 5⋅H_2_O; 0.9 g L^–1^ CoSO_4_, 7⋅H_2_O; 0.06 g L^–1^ BO_3_H_3_; 1 g L^–1^ Mo_7_O_24_ (NH_4_)_6_, 4⋅H_2_O; 0.04 g L^–1^ Ni(NO_3_)_2_, 6⋅H_2_O; 0.02 g L^–1^ Na_2_SeO_3_] adapted from Traore et al. (1983), 0.0001% β-Mercaptoethanol and buffered to pH 7.2 with NaOH. For proteomic analysis, *Dv*H were grown in 1 L Duran bottle containing 700 mL SKY medium.

### 2.2 Analytical methods

Total biomass concentrations (cell dry weight) were determined using 30 mL cell culture. Cells were harvested by centrifugation for 10 min at room temperature and the harvested cells were dried at 65°C under vacuum (Thermo Scientific Savant DNA120).

Organic acid quantification was determined by high-performance liquid chromatography (HPLC) using 1200 Infinity HPLC system (Agilent) equipped with a Hi-Plex H 300 mm × 7.7 mm column (Agilent). Samples of 25 μL that were previously clarified through 0.2 μm filters were applied to the columns equilibrated in 10 mmol L^–1^ H_2_SO_4_ with a flow of 0.6 mL min^–1^ at 50°C/30 min/RI detection (Agilent).

Hydrogen, carbon dioxide, and hydrogen sulfide contents in the gas phase were determined by gas chromatography (Agilent 4890D) equipped with a thermal conductivity detector. The gases were separated using a GS-CARBONPLOT column (30 m × 0.535 mm × 3.00 mm) using N_2_ at high purity as carrier gas. The injector temperature was 80°C, the oven temperature was 30°C and the detector temperature was 90°C.

Total sulfide ions content was determined based on colorimetric assay. First, the pH of the cell culture was increased by adding sodium hydroxide to 0.25 N final concentration. This aims at shifting the equilibrium toward the aqueous HS^–^ or S_2_^–^ forms which are more soluble than gaseous H_2_S. Besides, this also contributed to cell lysis, thus releasing intracellular sulfide ions. Following 30 min incubation at room temperature, samples were clarified through a 0.22 μm filter to remove cell debris, and 10–200 μL were used in the assay as described previously (Guiral et al., 2005).

### 2.3 Proteomic analysis

Samples for proteomic analysis were obtained by harvesting 1 L cell culture by centrifugation at 3000 × *g* for 20 min. The cell pellet (0.46 g wet weight) was resuspended with 10 mmol L^–1^ Tris–HCl pH 7.6, 5 mmol ethylenediaminetetraacetate (as buffer A) supplemented with protease inhibitor cocktail (SigmaFast tablets) and cells were broken by three passages through a cell disruptor (Constant system Ltd., Northants, UK) at 1.6 Kbar. Unbroken cells and cell debris were removed by centrifugation at 12,000 × *g*, 4°C, for 15 min, soluble and membrane fractions were separated by ultracentrifugation at 150,000 × *g*, 4°C for 45 min. Proteins (10 mg mL^–1^) from the membrane fraction were solubilized in buffer A, supplemented with 10% (v/v) glycerol and 1% (w/v) n-Dodecyl-β-D-maltoside at 4°C for 1 h. Unsolubilized proteins were removed by ultracentrifugation as described previously. The total proteins in the soluble and membrane fractions were identified by shotgun proteomics as described in Prioretti et al. (2023). Briefly, samples (40 μg) were loaded on a denaturing stacking gel (5% acrylamide), ran for approximately 5 min at 25 mA and 250 V, then stained with Coomassie blue. The protein band visible in the stacking gel was cut out from the gel and stored at −20°C before LC-MS/MS analysis. Stacking gels for soluble and membrane protein fractions were performed in triplicate, then the bands were digested by Trypsin/Lys C protease (Promega), after a classical double step of reduction and alkylation of cysteine amino acids. Tryptic peptides were analyzed on a Q-Exactive Plus mass spectrometer (Thermo Fisher Scientific) coupled to a nano-LC system (Ultimate 3000, Dionex) equipped with an EASY-spray column (PepMaP TM RSLC, C18, 2, 75 μm ID × 15 cm, Thermo Fisher Scientific), after a separation by a two step -linear gradient from 4 to 40% of mobile phase B [0.1% (vol/vol) formic acid (FA)/ 80% (vol/vol) acetonitrile] in mobile phase A [0.1% (vol/vol) FA] for 52 min. For peptide ionization in the nanosource spray, voltage was set at 1.9 kV and the capillary temperature at 275°C. Top 10 Data Dependent workflow was used in a 350–1900 m/z range and a dynamic exclusion of 30 s. Data were processed by Proteome Discoverer (Thermo Fisher, version: 2.4.1.15) using the Sequest HT algorithm with the search following settings : databank *Dv*H- (taxonomy ID 882) from Uniprot (version 2021-02-04); trypsin enzyme (maximum 2 missed cleavages); fixed modification: carbamidomethyl (Cys); variable modification: oxidation (Met); mass values specific for monoisotopic; precursor mass tolerance: ± 10 ppm; fragment mass tolerance: ± 0.02 Da. Peptide validation was based on score threshold at maximum Delta Cn 0.05. Proteins were identified if minimum 2 unique peptide sequences more than 6 amino acids passed the high confidence filter. For each replicate, results from soluble and membrane fractions were then combined as a single file. The mass spectrometry proteomics data will be deposited to the ProteomeXchange Consortium (Deutsch et al., 2023) via the PRIDE (Perez-Riverol et al., 2022) partner repository with the dataset identifier PXD046638.

### 2.4 Model reconstruction

Several steps were carried out to reconstruct the iDvu71 model. First, the considered metabolic network was reconstructed from the proteomic study performed in the present study and BiGG, KeGG and MetaNetx databases (Kanehisa, 2000; Ganter et al., 2013; King et al., 2016). The reconstructed metabolic model is presented in the Supplementary File 1 as a SBML file. To ensure standardized nomenclature and to facilitate comparison with other genome-scale metabolic models, all reactions and metabolites in the model were named with Kegg IDs.

Then, the network reconstruction was converted into a mathematical model and, finally, all simulations were carried out using the 3rd version of the Constraint-Based Metabolic Modeling Toolbox (COBRA Toolbox) (Heirendt et al., 2019) in the Matlab environment. Flux Balance Analysis (FBA) was employed as a method to predict the steady-state flux distribution in the metabolic network, by optimizing an objective function, typically biomass production or a specific metabolite production rate, subjected to constraints on the system’s available resources.

### 2.5 Mathematical determination of simulated concentrations based on simulated metabolic fluxes and model validation

Starting from the initial metabolic concentrations at 5.5 h, which corresponds to the beginning of the growth phase, the kinetics of metabolic concentrations were calculated using a time step of 0.01 h. For each time step, the concentration of each metabolite is updated using Euler’s method (Formula 1):


(1) $$C_{i}\,\left(t+\Delta \,t\right)=C_{i}\,\left(t\right)+\varphi_{i}^{F\,B\,A}\,\left(t\right)\,X\,\left(t\right)\,\Delta \,t$$

where *C*_*i*_(*t* + Δ*t*), in g L^–1^, is the predicted concentration of the *i^th^* metabolite at time t, *C*_*i*_(*t*) is the concentration of the metabolite at the current time step, $\varphi_{i}^{F\,B\,A}\,\left(t\right)$ is the *i^th^* metabolic flux simulated by FBA, *X*(*t*) is the biomass concentration as g L^–1^, at time t, and Δt is the time step (0.01 h).

The validation of iDvu71 was based on the comparison of simulated and experimental metabolite and biomass concentrations. To validate the model accuracy, the Pearson correlation coefficient was calculated between simulated and experimental data.

## 3 Results

### 3.1 Proteomic analysis

**FIGURE 1:**
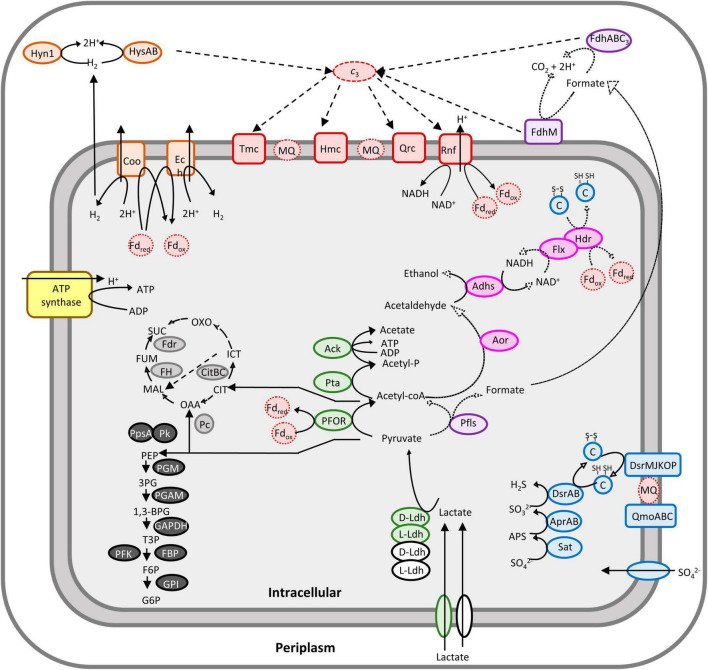
Overview of *Dv*H carbon and energy metabolisms under lactate/sulfate respiration. The pathways identified with high confidence by proteomic and metabolic analysis are shown in bold arrows. Pathways relying on the presence of enzyme identification by proteomic analysis only are shown in dashed arrows. Filling colors indicate different metabolic pathways: lactate utilization related to the *luo* genes cluster and orthologs in green and white, respectively; gluconeogenesis in black; uncomplete TCA cycle in gray; sulfate reduction in blue; hydrogen metabolism in orange; formate metabolism in purple; ethanol metabolism in pink; membrane and soluble electron transport systems in red. Letter code for each enzyme is reported in Table 1. Fd, ferredoxin; CIT, citrate; ICT, isocitrate; OXO, 2-oxoglutarate; OAA, oxaloacetate; MAL, malate; Fum, fumarate; Suc, succinate; PEP, phosphoenolpyruvate; 3PG, 3-phosphoglycerate; 1,3-BPG, 1,3-bisphosphoglycerate; T3P, triose-3-phosphate; G6P, glucose-6-phosphate; F6P, fructose-6-phosphate; Acetyl-P, acetyl phosphate; APS, adenosine-5′-phosphate.

To construct an accurate model of *Dv*H metabolism, proteomes from three independent cultures of *Dv*H grown to exponential phase (OD_600 *nm*_ ∼ 0.6) in SKY medium were analyzed by LC-MS/MS. Table 1 shows the results obtained for the combined soluble and membrane fractions of one biological replicate. Protein abundance was evaluated by spectral counting as illustrated by PSM (as peptide spectrum match) number for all replicates and listed in Supplementary Table 1. The genome of *Dv*H has 3351 predicted protein-encoding genes (Heidelberg et al., 2004), and a total of 1507–1669 unique proteins were detected in the three independent experiments corresponding to 42–47% genome coverage (Supplementary Table 1). Among them, attention was first paid toward the proteins involved in lactate metabolism, sulfate reduction and energy metabolism. All the key enzymes involved in the lactate oxidation in *Dv*H, which belongs to the gene cluster “*luo*” (DVU3025-33) (Wall et al., 2008; Vita et al., 2015) were detected with confidence (Table 1). In addition, two paralogs (DVU0390 and DVU0253) of the D-LDH located in the “*luo*” operon (Vita et al., 2015) and one ortholog of the L-LDH (DVU2789) from *Escherichia coli* (Dong et al., 1993) were also identified with confidence in these conditions (Table 1). Moreover, all proteins involved in the respiration of sulfate showed higher PSM number relative to those from other pathways indicating a higher global relative abondance (Table 1). This included two orthologs of sulfate permeases (DVU0279 and DVU0053), the sulfate adenylyltransferase (Sat), the adenylyl-sulfate reductase (AprAB), the dissimilatory sulfate reductase (DsrAB), the key sulfur transfer protein (DsrC), as well as the dissimilatory sulfite-reductase (DsrMKJOP) and the quinone- interacting oxidoreductase (QmoABC) complexes (Figure 1). Interestingly, both enzymes involved in the hydrogen- and formate-cycles were identified under these growth conditions (Figure 1). For instance, four different hydrogenases were identified in these samples (Table 1). This includes two membrane-bound [NiFe]-hydrogenases belonging to the *coo* and *ech* genes clusters and two periplasmic hydrogenases, the [NiFe]-hydrogenases (Hyn-1) and the [NiFeSe]-hydrogenase (Hys) (Wall et al., 2008; Keller and Wall, 2011; Baffert et al., 2019). While the expression of hydrogenases is thought to be constitutive in the cells (Ogata and Lubitz, 2021), the periplasmic Hys was found to be the most abundant periplasmic hydrogenase under these conditions, whereas the low PSM values for other hydrogenases suggested that they are considerably less abundant in the cells under these growth conditions (Table 1). Moreover, enzymes involved in formate metabolism in *Dv*H were also identified with confidence (Table 1). This included two isoforms of the PFLs (Keller and Wall, 2011), as well as subunits belonging to the membrane-associated FDH (FdhM) and the cytochrome *c*_3_-associated periplasmic FDH (FdhABC_3_) (Table 1).

**TABLE 1:** Tandem mass spectrometry of *Dv*H under lactate/sulfate respiration.

Enzymes	Subunit/ Protein	Accession	Locus tag	MW	PSM	Cov	Pep
**Lactate utilization**							
Pyruvate: ferredoxin oxidoreductase subunit A	PorA	Q72BR5	DVU_1569	61.8	13	23	8
Pyruvate: ferredoxin oxidoreductase subunit B	PorB	Q72BR4	DVU_1570	30.8	10	37	7
Pyruvate: ferredoxin oxidoreductase	PFOR	Q726T1	DVU_3025	30.8	402	37	7
L-lactate permease	Ltp	Q726T0	DVU_3026	60.8	6	13	6
D-lactate dehydrogenase subunit A	D-LdII-A	Q726S9	DVU_3027	49.3	85	81	32
D-lactate dehydrogenase subunit B	D-LdII-B	Q726S8	DVU_3028	45.9	32	45	16
Phosphate acetyl transferase	Pta	Q726S7	DVU_3029	76.8	121	75	41
Acetate kinase	Ack	Q726S6	DVU_3030	44.1	77	60	28
Conserved hypothetical protein		Q726S5	DVU_3031	39.0	12	27	8
L-lactate dehydrogenase subunit A	LldG	Q726S4	DVU_3032	22.6	21	63	11
L-lactate dehydrogenase subunit B	LldH	Q726S3	DVU_3033	79.6	106	63	43
Lactate dehydrogenase		P62051	DVU_0600	32.2	2	13	2
D-lactate dehydrogenase subunit A (Dld-II family)		Q72DV3	DVU_0826	47.4	2	4	2
D-lactate dehydrogenase subunit B (Dld-II family)		Q72DV2	DVU_0827	50.1	7	22	6
D-lactate dehydrogenase (Dld-II family)		Q72F25	DVU_0390	48.2	25	51	17
D-lactate dehydrogenase (Dld-II family)		Q72FG1	DVU_0253	103	88	62	52
L-lactate dehydrogenase (LldG family)		Q72B57	DVU_1781	23.9	–	–	–
L-lactate dehydrogenase (LldH family)		Q72B56	DVU_1782	52.6	4	8	4
Lactate permeases, putative		Q72B55	DVU_1783	27.4	3	14	2
		Q72A87	DVU_2110	57.9	5	9	3
		Q729R4	DVU_2285	53.0	5	13	5
		Q725Z0	DVU_3284	57.7	–	–	–
**Sulfate reduction**							
Sulfate permeases		Q72FD5	DVU_0279	60.6	9	21	8
		Q72G10	DVU_0053	68.1	5	14	4
Sulfate adenylyl transferase	Sat	Q72CI8	DVU_1295	47.4	301	87	49
Adenylyl sulfate reductase, AprAB complex, subunit B	AprB	Q72DT3	DVU_0846	18.5	47	81	8
Adenylyl sulfate reductase, AprAB comple, subunit A	AprA	Q72DT2	DVU_0847	74.6	545	80	61
Sulfite reductase, DsrABC complex, subunit A	DsrA	P45574	DVU_0402	49.1	153	74	37
Sulfite reductase, DsrABC complex, subunit B	DsrB	P45575	DVU_0403	42.5	117	59	28
Sulfite reductase, DsrABC complex, subunit D	DsvD	Q46582	DVU_0404	8.8	7	60	3
Sulfite reductase, DsrABC complex, subunit C	DsrC	P45573	DVU_2776	11.9	14	66	7
Quinone oxidoreductase, QmoABC complex, subunit A	QmoA	Q72DT1	DVU_0848	44.6	82	78	28
Quinone oxidoreductase, QmoABC complex, subunit B	QmoB	Q72DT0	DVU_0849	82.5	211	85	54
Quinone oxidoreductase, QmoABC complex, subunit C	QmoC	Q72DS9	DVU_0850	42.6	46	42	18
Quinone oxidoreductase, QmoABC complex, hypothetical protein	QmoD	Q72DS8	DVU_0851	25.8	63	86	17
Dissimilatory sulfite reductase, DsrMKJOP complex, subunit P	DsrP	Q72CJ7	DVU_1286	43.5	–	–	–
Dissimilatory sulfite reductase, DsrMKJOP complex, subunit O	DsrO	Q72CJ6	DVU_1287	29.0	28	76	17
Dissimilatory sulfite reductase, DsrMKJOP complex, subunit J	DsrJ	Q72CJ5	DVU_1288	14.5	–	–	–
Dissimilatory sulfite reductase, DsrMKJOP complex, subunit K	DsrK	Q72CJ4	DVU_1289	60.7	40	54	20
Dissimilatory sulfite reductase, DsrMKJOP complex, subunit M	DsrM	Q72CJ3	DVU_1290	38.1	30	37	13
**Hydrogen metabolism**							
Membrane-bound hydrogenase Ech complex, subunit A	EchA	Q72EY4	DVU_0434	69.1	4	8	3
Membrane-bound hydrogenase Ech complex, subunit B	EchB	Q72EY5	DVU_0433	31.2	–	–	–
Membrane-bound hydrogenase Ech complex, subunit C	EchC	Q72EY6	DVU_0432	16.8	–	–	–
Membrane-bound hydrogenase Ech complex, subunit D	EchD	Q72EY7	DVU_0431	14.6	2	40	2
Membrane-bound hydrogenase Ech complex, subunit E	EchE	Q72EY8	DVU_0430	40.1	5	18	5
Membrane-bound hydrogenase Ech complex, subunit F	EchF	Q72EY9	DVU_0429	14.8	2	19	2
Membrane-bound hydrogenase Coo complex, subunit M	CooM	Q729R3	DVU_2286	131.0	12	12	11
Membrane-bound hydrogenase Coo complex, subunit K	CooK	Q729R2	DVU_2287	33.7	7	8	3
Membrane-bound hydrogenase Coo complex, subunit L	CooL	Q729R1	DVU_2288	15.7	3	24	2
Membrane-bound hydrogenase Coo complex, subunit X	CooX	Q729R0	DVU_2289	22.7	6	21	3
Membrane-bound hydrogenase Coo complex, subunit U	CooU	Q729Q9	DVU_2290	19.6	5	27	4
Membrane-bound hydrogenase Coo complex, subunit H	CooH	Q729Q8	DVU_2291	40.7	25	70	19
Hydrogenase nickel-insertion protein	HypA	Q729Q7	DVU_2292	12.7	–	–	–
Membrane-bound hydrogenase Coo complex, subunit F	CooF	Q729Q6	DVU_2293	19.0	–	–	–
[FeFe]-hydrogenase Hyd complex, subunit L	HydL	P07598	DVU_1769	45.9	–	–	–
[FeFe]-hydrogenase Hyd complex, subunit S	HydS	P07603	DVU_1770	13.6	–	–	–
[NiFeSe]-hydrogenase Hys complex, subunit B	HysB	Q72AS4	DVU_1917	33.9	15	53	12
[NiFeSe]-hydrogenase Hys complex, subunit A	HysA	Q72AS3	DVU_1918	55.8	96	67	33
[NiFe]-hydrogenase Hyn1 complex, subunit B	HynB1	Q06173	DVU_1921	34.2	–	–	–
[NiFe]-hydrogenase Hyn1 complex, subunit A	HynA1	Q72AS0	DVU_1922	62.7	25	34	15
[NiFe]-hydrogenase Hyn2 complex, subunit B	HynB2	P61429	DVU_2525	34.5	–	–	–
[NiFe]-hydrogenase Hyn2 complex, subunit A	HynA2	Q728S7	DVU_2526	61.2	–	–	–
**Formate metabolism**							
Pyruvate formate lyase activating enzyme, putative		Q729S8	DVU_2271	32.4	–	–	–
Pyruvate formate lyase, putative		Q729S7	DVU_2272	91.2	13	15	9
Pyruvate formate lyase, putative		Q727N1	DVU_2824	93.9	28	35	22
Pyruvate formate lyase activating enzyme, putative		Q727N0	DVU_2825	33.7	–	–	–
Formate dehydrogenase FdhABC_3_ complex, cytochrome *c*_3_ subunit	Fdhc_3_	Q727P6	DVU_2809	15.8	–	–	–
Formate dehydrogenase formation protein	FdhE	Q727P5	DVU_2810	29.6	–	–	–
Formate dehydrogenase FdhABC_3_ complex, subunit B	FdhB	Q727P4	DVU_2811	24.5	2	10	1
Formate dehydrogenase FdhABC_3_ complex, subunit A	FdhA	Q727P3	DVU_2812	113.3	52	47	34
Formate dehydrogenase FdhAB complex, subunit α	Fdhα	Q72EJ1	DVU_0587	111.2	–	–	–
Formate dehydrogenase FdhAB complex, subunit β	Fdhβ	Q72EJ0	DVU_0588	26.4	–	–	–
Formate dehydrogenase FdhM complex, subunit B	CfdB	Q728X1	DVU_2481	29.2	2	16	2
Formate dehydrogenase FdhM complex, subunit A	CfdA	Q728X0	DVU_2482	110.8	48	43	30
Formate dehydrogenase FdhM complex, cytochrome *c* putative	CfdE	Q728W9	DVU_2483	61.9	11	23	8
Formate dehydrogenase FdhM complex, cytochrome *c* putative	CfdD	Q728W8	DVU_2484	47.7	7	13	3
**Transmembrane electron transport complexes**							
Quinone reductase complex QrcDCBA, subunit D	QrcD	Q72E86	DVU_0692	47.5	8	16	6
Quinone reductase complex QrcDCBA, subunit C	QrcC	Q72E85	DVU_0693	29.0	16	39	8
Quinone reductase complex QrcDCBA, subunit B	QrcB	Q72E84	DVU_0694	72.3	105	81	36
Quinone reductase complex QrcDCBA, subunit A	QrcA	Q72E83	DVU_0695	7.4	–	–	–
Transmembrane complex TmcABCD, subunit A	TmcA	Q72FF1	DVU_0263	13.5	–	–	–
Transmembrane complex TmcABCD, subunit B	TmcB	Q72FF0	DVU_0264	49.6	46	51	21
Transmembrane complex TmcABCD, subunit C	TmcC	Q72FE9	DVU_0265	24.0	8	19	4
Transmembrane complex TmcABCD, subunit D	TmcC	Q72FE8	DVU_0266	44.9	19	43	11
High molecular weight cytochrome complex Hmc, protein 6	HmcF	P33393	DVU_0531	52.7	29	43	17
High molecular weight cytochrome complex Hmc, protein 5	HmcE	P33392	DVU_0532	25.3	–	–	–
High molecular weight cytochrome complex Hmc, protein 4	HmcD	P33391	DVU_0533	5.8	–	–	–
High molecular weight cytochrome complex Hmc, protein 3	HmcC	P33390	DVU_0534	43.1	2	6	2
High molecular weight cytochrome complex Hmc, protein 2	HmcB	P33389	DVU_0535	40.0	18	47	14
High molecular weight cytochrome complex Hmc, protein 1	HmcA	P24092	DVU_0536	58.9	12	26	16
Cytochrome c	DhcA	Q727R4	DVU_2791	27.8	–	–	–
Rnf complex, subunit C	RnfC	Q727R3	DVU_2792	43.5	26	60	14
Rnf complex, subunit D	RnfD	Q727R2	DVU_2793	33.7	–	–	–
Rnf complex, subunit G	RndG	Q727R1	DVU_2794	20.1	8	49	7
Rnf complex, subunit E	RnfE	Q727R0	DVU_2795	23.8	5	17	3
Rnf complex, subunit A	RnfA	Q727Q9	DVU_2796	20.8	–	–	–
Rnf complex, subunit B	RnfB	Q727Q8	DVU_2797	30.9	4	18	4
Membrane associated lipoprotein	ApbE	Q727Q7	DVU_2798	35.0	–	–	–
**Ethanol metabolism**							
Aldehyde oxidoreductase	Aor	Q72BS5	DVU_1559	97.4	66	42	30
Iron-containing alcohol dehydrogenases	Adh1	Q729E6	DVU_2405	41.7	111	64	27
		Q729Z6	DVU_2201	41.9	37	52	17
		Q72F61	DVU_0353	39.4	24	54	15
		Q727H0	DVU_2885	42.0	7	25	5
		Q727F0	DVU_2905	41.0	3	16	3
	Adh2	Q729F5	DVU_2396	41.0	2	6	2
		Q728Q8	DVU_2545	40.0	–	–	–
NADH oxidoreductase complex Flx, subunit A	FlxA	Q729F2	DVU_2399	31.0	24	64	13
NADH oxidoreductase complex Flx, subunit B	FlxB	Q729F1	DVU_2400	39.4	20	35	11
NADH oxidoreductase complex Flx, subunit CD	FlxCD	Q729F0	DVU_2401	54.1	46	74	22
Hetero disulfide reductase complex Hdr, subunit A	HdrA	Q729E9	DVU_2402	70.9	52	50	25
Hetero disulfide reductase complex Hdr, subunit B	HdrB	Q729E8	DVU_2403	35.1	17	45	10
Hetero disulfide reductase complex Hdr, subunit C	HdrC	Q729E7	DVU_2404	21.0	6	25	4
**Gluconeogenesis**							
Pyruvate kinase	Pk	Q728T8	DVU_2514	50.4	31	55	17
Phosphoenolpyruvate synthase	PpsA	Q72B07	DVU_1833	132.6	184	68	85
Pyruvate carboxylase	Pc	Q72B06	DVU_1834	136.3	111	53	55
2,3 bisphosphoglycerate-independent phosphoglycerate mutase	PGM	Q72BL6	DVU_1619	54.1	8	24	7
Phosphoglycerate kinase	PGAM	P62412	DVU_2529	414	41	62	18
Glyceraldehyde-3-phosphate dehydrogenases	GAPDH	Q72EL3	DVU_0565	37.0	28	67	18
	GAPDH	Q72A53	DVU_2144	35.6	27	58	15
Fructose bi-phosphate aldolase	FBP	Q72A54	DVU_2143	33.5	22	62	14
6-phosphofructokinase	PFK	Q72AD6	DVU_2061	48.1	20	42	12
Glucose 6-phosphate isomerase	GPI	Q725H4	DVU_3222	48.7	18	38	13
**TCA cycle**							
Aconitate hydratase	CitB	Q72D65	DVU_1064	67.5	49	54	23
Isocitrate dehydrogenase	CitC	Q72EU1	DVU_0477	41.5	24	53	17
Fumarate reductase (Fdr) subunit C	FdrC	Q726D0	DVU_3261	23.7	–	–	–
Subunit A	FdrA	Q726C9	DVU_3262	65.9	19	30	14
Subunit B	FdrB	Q726C8	DVU_3263	27.8	5	13	3
Fumarate hydratase	FH	Q726A9	DVU_3264	29.5	2	15	2
Tartrate dehydratase		Q726A8	DVU_3265	19.4	–	–	–

Proteins were identified from the soluble and membrane fractions of *Dv*H grown to exponential phase under anaerobic conditions in SKY medium as described in the section “2 Materials and methods.” Results corresponding to one of three independent experiments were reported in this table. Protein abundance (indicated by spectral counting as PSM) for all replicates is reported in Supplementary Table 1. Accession: accession number in the UniProt database. MW: theoretical molecular weight in kDa. PSM: peptide spectrum match number (given by the algorithm and corresponding to the total number of identified peptide sequences for the protein, including those redundantly identified). Cov: protein sequence coverage by the matching peptides in percentage. Pep: number of distinct peptides matching the protein sequence and unique to this protein. -: not identified by mass spectrometry.

In both hydrogen- and formate-cycling model, electrons released in the periplasm are cycling back to the cytoplasm through transmembrane electron transport complexes, thus providing electrons for sulfate reduction and energy conservation in *Dv*H (Keller and Wall, 2011; Tang et al., 2021). Among them, the quinone reductase complex (QrcDCBA) (Venceslau et al., 2010) and the transmembrane complex (TmcABCD) were identified with high confidence (Table 1) in these samples. Moreover, the high molecular weight cytochrome (HmcBCDE) (Venceslau et al., 2010) and the Rnf complex, which most likely acts as proton-pumping ferredoxin:cyt *c*_3_ oxidoreductase or ferredoxin:NAD^+^ oxidoreductase (Venceslau et al., 2010), were also detected by proteomic analysis (Table 1). Altogether, these results suggested that both hydrogen- and formate-cycles operate simultaneously during lactate oxidation under these conditions (Figure 1). Nevertheless, it is important to note that no transient formate was detected by HPLC in these samples (Supplementary Figure 1) by contrast with hydrogen during the various growth phases. The absence of formate in the cell supernatant could be explained by the fact that formate was metabolized straight after being produced at significant rate, or not excreted by the cell, or below the HPLC detection limit.

Strikingly, proteomic analysis revealed the presence of six alcohol dehydrogenases (Adhs), with Adh1 (DVU2405) being the most abundant (Table 1). This is in in line with previous studies showing that DVU2405 is one of the most highly expressed genes in cells grown in lactate/sulfate medium (Haveman et al., 2003; Zhang et al., 2006). It is assumed that Adh1 could function with the Hdr-Flx complex, identified with high confidence in these samples too (Table 1). The Hdr-Flx complex is formed by two sub-complexes: an HdrABC-like components involved in “flavin-based electron bifurcation/confurcation” linked to a flavin-dependent oxidoreductase named FlxABCD (Appel et al., 2021). It was proposed that during ethanol/sulfate growth, the NADH formed during ethanol oxidation by Adh1 is reoxidized via electron bifurcation by the Hdr-Flx complex to ferredoxins and a second electron acceptor, most likely DsrC (Ramos et al., 2015). By contrast, during fermentation Hdr-Flx is assumed to be involved in NAD^+^ recycling via electrons confurcation from reduced ferredoxins and the reduced DsrC dithiol. The NADH formed through this reaction would serve as reducing equivalent for the reduction of acetaldehyde to ethanol by Adh1 (Ramos et al., 2015). Given that no ethanol was detected by HPLC in the cell culture supernatant, here we propose that ethanol produced from acetyl-coA could be oxidized by Adh1 while producing NADH, which is used as electron donor for the reduction of the ferredoxin pool and DsrC through “flavin-based electron bifurcation” by Hdr-Flx, thus providing additional reducing equivalent for sulfate reduction (Figure 1). This is in line with previous studies on *Desulfovibrio alaskensis G20* that pointed toward the involvement of Hdr-Flx complex in pyruvate/sulfate respiration, although it is not essential for growth (Meyer et al., 2014).

### 3.2 Reconstruction of the iDvu71 model

By combining the proteomic data obtained in the present study to information from BiGG, KeGG, MetaNetx databases, a simplified metabolic model called iDvu71 was reconstructed. The main metabolic pathways considered in this model were gluconeogenesis, fermentative pathways, and the uncomplete TCA cycle. The addition of the pentose phosphate pathway was carried out based on ^13^C tracing experiments (Tang et al., 2007). Dissimilatory sulfate reduction, taking place in several steps (Sim et al., 2017), from the cytoplasmic reduction of sulfate to the production of hydrogen sulfide was also included in the model (Voordouw, 1995; Sim et al., 2017; Baffert et al., 2019; Zhu et al., 2019). Based on the proteomic analysis conducted in the present study, the ethanol pathway including alcohol dehydrogenases (Adhs) and aldehyde dehydrogenase (Aor) that may be related to the acetate synthesis (Pereira et al., 2008) was also integrated. The addition of all these metabolic reactions enlarged the diversity of metabolites that can be consumed by *Dv*H. Once reconstructed, iDvu71, was composed of 71 chemical reactions and 81 metabolites.

### 3.3 iDvu71 validation

**FIGURE 2:**
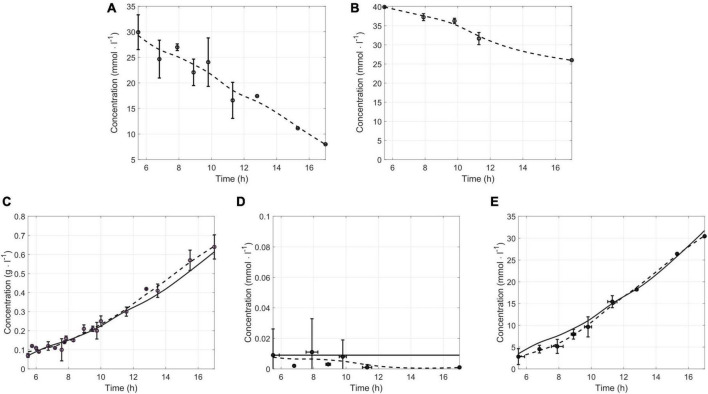
Validation of iDvu71 model. Lactate and sulfate concentrations (mmol L^–1^) measured experimentally and interpolation of these concentrations are given in **(A,B)**, respectively. Comparison of experimental (*n* ≥ 3) and simulated concentrations of *Dv*H cells [**(C)**, in g L^–1^], hydrogen [**(D)**, in mmol L^–1^] and acetate [**(E)** in mmol L^–1^]. The round points (o) correspond to the measured experimental concentration, the dashed line (–) corresponds to the experimental interpolation, and the solid line (-) to the simulated concentration.

A comparison of simulated and measured concentrations was performed to validate the iDvu71 model. Constraints such as consumption fluxes of lactate and sulfate were imposed. These calculated fluxes, based on sulfate and lactate concentrations determined throughout growth of *Dv*H in SKY medium (Figures 2A, B) were, 5 mmol g_*DCW*_^–1^ h^–1^ and 2.5 mmol g_*DCW*_^–1^h^–1^, respectively. These fluxes enable the determination of simulated concentrations of biomass, acetate and hydrogen which have also been experimentally determined (Figures 2C–E). It should be noted that, except acetate, no other organic acid was produced and secreted by *Dv*H throughout the entire growth (Supplementary Figure 1). Finally, simulations were carried out for various time points, relying on the interpolation of experimental measurements to provide more accurate and consistent results. A good Pearson correlation was obtained between the experimental and simulated concentrations (Figures 2C–E). Indeed, correlations higher than 0.95 for each metabolite between experimental and simulated concentrations were obtained, validating iDvu71 model.

### 3.4 Influence of sulfate on hydrogen production/consumption by *Dv*H

**FIGURE 3:**
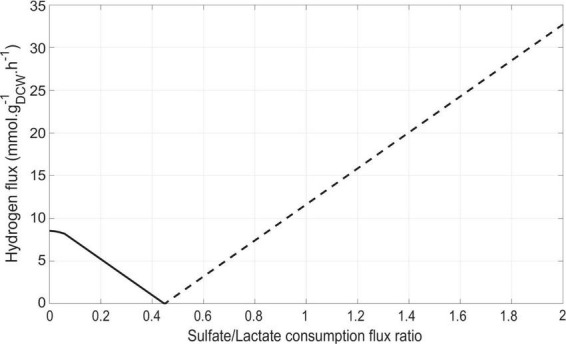
Impact of the sulfate/lactate flux ratio on hydrogen flux. Lactate consumption was set at a constant flux of 5 mmol g_DCW_^–1^ h^–1^. The hydrogen production and consumption fluxes are shown by solid (-) and dashed (–) lines, respectively.

After validation of iDvu71 model, simulations were run with (i) the “objective function” being the maximization of the biomass synthesis flux, (ii) a constant average lactate consumption flux of 5 mmol g_*DCW*_^–1^ h^–1^, and (iii) a gradually increased rate of sulfate consumption, starting from zero and increasing by 0.1 mmol g_*DCW*_^–1^ h^–1^ per simulation run. Under these conditions, the ratio between the sulfate consumption flux and the lactate consumption flux varied from 0 to 2 (Figure 3). Interestingly, a significant shift in the behavior of *Dv*H hydrogen metabolism was observed when this ratio reached 0.45. Below this value, hydrogen was produced by *Dv*H whereas above it, hydrogen was utilized as energy source. Because sulfate respiration is associated with a net consumption of two ATP per sulfate, two molecules of lactate are required per molecule of reduced sulfate yielding to zero net ATP through substrate-level phosphorylation. Further oxidation of the hydrogen resulting from the lactate oxidation to pyruvate and pyruvate oxidation to acetyl-coA could contribute to the generation of protomotive force while providing the electrons required for sulfate respiration. Hence, hydrogen consumption flux increased with the concentration of sulfate in the environment. Instead, under sulfate limitation, the excess of reducing equivalents is dissipated through the production of hydrogen. However, under these conditions cell growth is regulated by the H_2_-partial pressure and occurs mostly when co-cultured with H_2_-scavenging microorganisms (Bryant et al., 1977). Therefore, the hydrogen production flux is difficult to determine experimentally under fermentative growth and can only be accessed through metabolic modeling in these conditions. This emphasizes the importance of developing accurate models to capture hidden metabolic fluxes. From these simulations, the sulfate/lactate consumption flux ratio could be considered as a relevant indicator of *Dv*H energy metabolism. Besides, our simulations are in line with previous studies using *Solidesulfovibrio fructosivorans*, showing that the gaseous H_2_ content strongly depends on the amount of sulfate in the medium (Payne et al., 2022; Kpebe et al., 2023).

**FIGURE 4:**
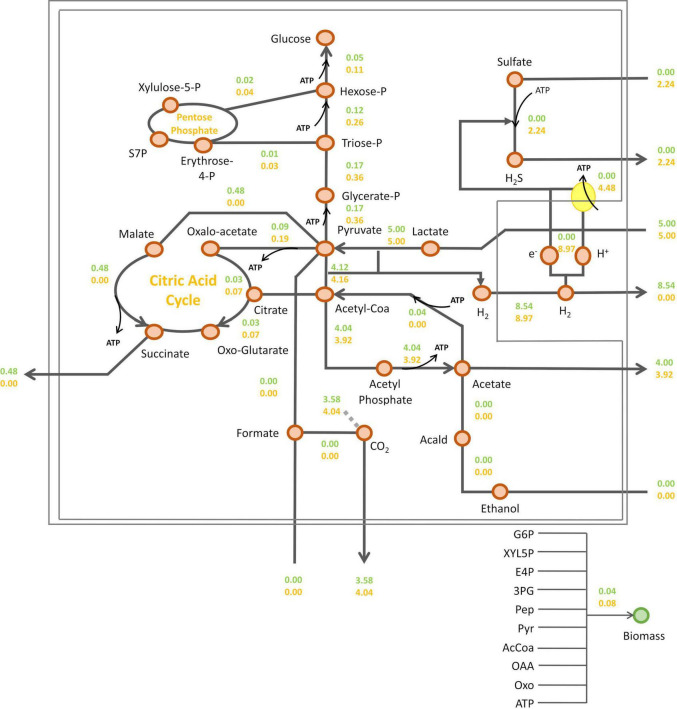
Prediction of *Dv*H intracellular fluxes by iDvu71. Lactate consumption flux was set at 5 mmol g_DCW_^–1^ h^–1^ and a sulfate consumption flux of 2.24 mmol g_DCW_^–1^ h^–1^, corresponding to a sulfate/lactate flux ratio of 0.45 (orange) or without sulfate consumption (green). All metabolic fluxes were expressed in mmol g_DCW_^–1^ h^–1^ except biomass synthesis flux which was expressed in g_DCW_ g_DCW_^–1^ h^–1^. Acald: acetaldehyde; AcCoA, acetyl-coA; Pyr: pyruvate; PEP, phosphoenolpyruvate; 3PG, 3-phosphoglycerate; E4P, erythrose-4-phosphate; XYL5P, xylulose-5-phosphate; G6P, Glucose-6-phosphate; S7P, sedoheptulose-7-phosphate; OXO, 2-oxoglutarate; OAA, oxaloacetate.

Then, intracellular fluxes were simulated at the metabolic shift, i.e., a sulfate consumption flux/lactate consumption flux ratio of 0.45, or in the absence of sulfate (Figure 4). As expected, sulfate respiration was abolished in the absence of sulfate. As a consequence, the electrons generated from pyruvate oxidation by the pyruvate-ferredoxin oxidoreductase (PFOR), were transferred to hydrogenases leading to hydrogen synthesis by *Dv*H (Figure 4). According to this simulation, succinate may be produced in the absence of sulfate (Figure 4). It was proposed that during fermentation of organic acids, part of the pyruvate is converted through reductive carboxylation to fumarate, which is then used as electron acceptor and reduced to succinate (Rabus et al., 2015). This is in line with previous studies in *D. alaskensis* G20 showing that succinate accumulate in the cell suspension under fermentative, sulfate-limited respiratory and co-culture conditions, while no succinate was detected in the cell suspension in excess of sulfate (Meyer et al., 2014). The addition of sulfate in the medium resulted in a twofold increase in fluxes leading to biomass synthesis (from 0.04 to 0.08 g_*CDW*_ g_*CDW*_^–1^ h^–1^). Indeed, sulfate respiration leads to the production of protons gradient resulting in ATP synthesis through ATP synthase (Pohorelic, 2001). This is illustrated by a proton flux of 8.97 mmol g_*DCW*_^–1^ h^–1^ in the presence of sulfate. Such significant increase in ATP production occurring in the presence of sulfate in the environment, resulted in a rewiring carbon flux toward gluconeogenesis. This is illustrated by a twice increase in the gluconeogenesis flux that led to glucose-6-phosphate (G6P), enhancing the synthesis of biomass precursors. In this condition, the succinate production was abolished (Figure 4).

### 3.5 Impact of hydrogen and formate-cycling mechanisms on biomass and hydrogen synthesis

**FIGURE 5:**
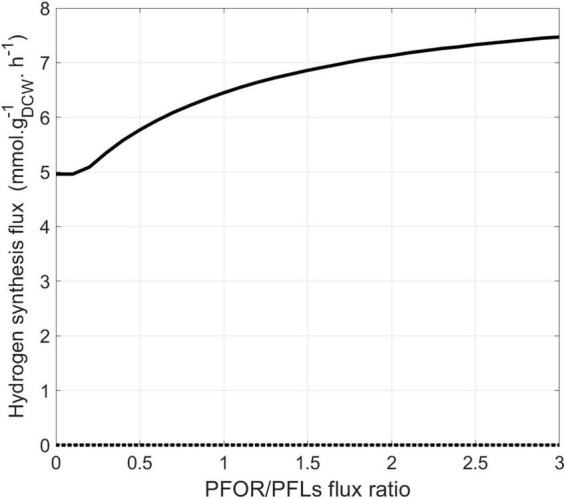
Model prediction for hydrogen synthesis flux (mmol g_DCW_^–1^ h^–1^) depending on the PFOR/PFLs flux ratio. Hydrogen intracellular flux was simulated in the presence (dashed line) or absence (solid line) of sulfate.

**FIGURE 6:**
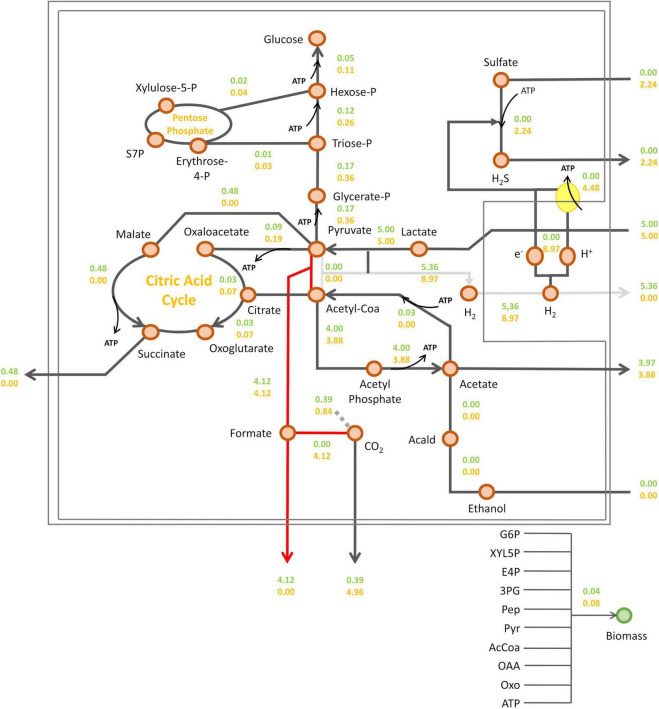
Prediction of *Dv*H intracellular fluxes under constrained PFOR/PFLs ratio at 0. Fluxes were calculated with a lactate consumption flux set at 5 mmol g_DCW_^–1^ h^–1^ and a sulfate consumption flux at 2.24 mmol g_DCW_^–1^ h^–1^, corresponding to a sulfate/lactate flux ratio of 0.45 (orange) or in sulfate-free condition (green). All metabolic fluxes were expressed in mmol g_DCW_^–1^ h^–1^ except biomass synthesis flux which was expressed in g_DCW_ g_DCW_^–1^ h^–1^. The red and gray arrows indicate the main changes in D*vH* metabolism due to the constrained PFOR/PFLs ratio compared to simulation with unconstrained PFOR/PFLs ratio reported in Figure 4: in red, the formate excretion and in gray, the reduced hydrogen synthesis. Acald, acetaldehyde; AcCoA, acetyl-coA; Pyr, pyruvate; PEP, phosphoenolpyruvate; 3PG, 3-phosphoglycerate; E4P, erythrose-4-phosphate; XYL5P, xylulose-5-phosphate; G6P, Glucose-6-phosphate; S7P, sedoheptulose-7-phosphate; OXO, 2-oxoglutarate; OAA, oxaloacetate.

In the previous set of simulations, no formate synthesis was simulated. Indeed, unlike hydrogen, no transient formate was experimentally detected in the cell culture supernatant under these growth conditions. However, as previously mentioned, some studies have highlighted the role of formate metabolism in energy conservation in *Dv*H. Additionally, two alternative pathways for the interconversion of pyruvate to acetyl-coA were identified by proteomic analysis (Figure 1). This includes the PFOR pathway (which simultaneously produces reduced ferredoxins) and the PFLs pathway (which leads to the production of formate as a by-product). To assess the respective impact of these metabolic pathways on biomass and hydrogen synthesis, a new series of simulations was performed. During these, the metabolic fluxes of, respectively, PFOR and PFLs were imposed in two different conditions (i) without sulfate or (ii) with a sulfate/lactate consumption flux ratio of 0.45. For both conditions, a PFOR/PFLs flux ratio ranging from 0 (as no flux through PFOR) to 3 (PFOR flux was threefold higher than the PFLs one) was applied. The influence of formate production via the PFLs pathway on hydrogen evolution for both conditions is depicted in Figure 5. As expected, hydrogen was produced by *Dv*H without sulfate in the culture medium. But in this condition, hydrogen production varied depending on the ratio between the PFOR and PFLs fluxes from 4.96 mmol g_*DCW*_^–1^ h^–1^ to 7.48 mmol g_*DCW*_^–1^ h^–1^ for a PFOR/PFLs ratio increasing from 0 to 3, respectively (Figure 5). This suggests that although both pathways can contribute to hydrogen production in *Dv*H, it is favored through the PFOR pathway. Further simulations were performed when the PFOR activity was completely abolished (PFOR/PFLs ratio of 0) and in the absence or presence of sulfate (Figure 6). According to these simulations, the biomass synthesis flux remained unchanged whatever the presence of electrons acceptor (0.04 g_*DCW*_ g_*DCW*_^–1^ h^–1^ in the absence of sulfate or 0.08 g_*DCW*_ g_*DCW*_^–1^ h^–1^ for a sulfate/lactate consumption flux ratio of 0.45) (Figure 6). In fact, the results of the two simulations clearly showed that, the PFLs flux reached the same value, 4.12 g_*DCW*_^–1^ h^–1^ (Figure 6) than the one simulated for PFOR flux (Figure 4) when no constraint was imposed on the iDvu71 model to use one pathway or the other. Moreover, as the stoichiometry of both PFOR and PFLs reaction is the same (1 pyruvate for 1 acetyl-coA), no significant change was observed in downstream reactions leading to acetate and succinate synthesis (Figures 4, 6). Finally, the hydrogen flux in absence of sulfate was reduced by 40%, from 8.54 mmol g_*DCW*_^–1^ h^–1^ (Figure 4) to 5.36 mmol g_*DCW*_^–1^ h^–1^ (Figure 6). This most likely resulted from rerouting of electrons toward formate synthesis, which is accompanied by a significant increase in formate production from 0 (Figure 4) to 4.12 mmol g_*DCW*_^–1^ h^–1^ (Figure 6).

## 4 Discussion

In the past, several genomic, transcriptomic, or proteomic studies have provided important insights into bioenergetics pathways of SRMs using *Dv*H as model organism (Heidelberg et al., 2004). Hence, several mechanisms were previously proposed to explain *Dv*H bioenergetics based on the identification and quantification of transcripts and enzyme products under various growth conditions (Heidelberg et al., 2004; Zhang et al., 2006; Pereira et al., 2008; Keller and Wall, 2011). Despite these studies, the mechanism of energy conservation in *Dv*H is far from being completely understood. One reason for this is that *Dv*H exhibit high redundancy of enzymes (e.g., hydrogenases). It is still not clear whether each enzyme has a specialized function or if it allows functional substitution depending on environmental conditions (Baffert et al., 2019). Hence, FBA can provide a comprehensive picture of metabolic network in the cell. Three metabolic models for this organism were previously proposed. The two first (Stolyar et al., 2007; Zomorrodi and Maranas, 2012), which are closely related and can be considered as simplified models, were reconstructed to study the impact of *Dv*H presence in syntrophy with *Methanococcus maripaludis* S2. However, when applied to a *Dv*H pure culture, the simulations were not satisfactory (data not shown). Using the model from Stolyar et al. (2007), no solution was reached with the constraints that were imposed to the model. This was attributed to gaps in the metabolic network reconstructed in Stolyar work. This model was completed by Zomorrodi and Maranas (2012) who introduced some metabolic pathways such as transfer equations between extracellular compartment and the cytosol, for instance. Simulations with this model were quite satisfactory regarding biomass synthesis (0.85 correlation). However, the acetate synthesis was always underestimated by a factor 2. By carefully checking the carbon fluxes (consumption and production fluxes), it was noticed that 20% of consumed carbon was not recovered in the carbon products. This led to an unmet global carbon mass balance. The most recent model, the iJF744 model (Flowers et al., 2018), is a genome-based model. Such an approach may suffer a lack of traceability and reproducibility in the model reconstruction (Heavner and Price, 2015). Moreover, with the constraints applied in the present study, using iJF744 model, the *Dv*H biomass concentration was overestimated whereas the acetate synthesis was underestimated. To resolve this divergence between experimental and simulated results, some modifications were performed in the metabolic network used by Flowers et al. (2018). The synthesis of formate by Formyltetrahydrofolate amidohydrolase, as proposed in the model, was replaced by the oxidation of pyruvate into formate as experimentally demonstrated by Zhou et al. (2017). This change induced a reduction in the biomass synthesis flux and an increase in acetate synthesis flux. However, even improved, the simulated fluxes remained unsatisfactory. For this reason, the choice was made to reconstruct a new transparent model, iDvu71, combined to an original proteomic analysis to investigate the role of hydrogen and formate metabolism in *Dv*H metabolism.

The proteomic analysis was conducted using *Dv*H grown to exponential phase in a medium containing lactate as carbon and energy source and sulfate as electron acceptor (SKY medium). This study provided an overall view of the bioenergetic metabolism of sulfate respiration in *Dv*H (Figure 1). These data are consistent with previous proposed mechanisms based on proteomic and transcriptomic analysis under similar growth conditions (Zhang et al., 2006; Pereira et al., 2008). Especially, all the enzymes involved in the lactate utilization machinery (encoded within the *luo* gene cluster) as well as few LDHs orthologs and the enzymes involved in dissimilatory sulfate reduction were identified with high confidence in these samples (Table 1). Notably, the PFOR (DVU_3025), the Apr subunit A (DVU_0847) and sat (DVU_1295) are ones of the most abundant proteins (Supplementary File 2). Interestingly, among the four hydrogenases identified in these samples, the periplasmic HysAB was the most abundant while the FeFe-hydrogenase (Hyd) and the NiFe-hydrogenase isoform-2 (Hyn-A2) could not be identified in the three different replicates (Supplementary Table 1). These results differed from previous proteomic study, where the periplasmic NiFe-hydrogenase isoform-1 (Hyn-A1) was found to be the most abundant hydrogenase, while Hys was not even detected (Zhang et al., 2006). Such discrepancy might result from variation in the expression level of the different hydrogenases depending on metals availability in the growth medium. Indeed, it was shown that the Hys hydrogenase is dominant when nickel and selenium are available (Valente et al., 2006). Hence, this result does not particularly reflect a specialized role for the periplasmic Hys hydrogenase under these conditions. Instead, it greatly illustrated the high versatility of *Dv*H energy metabolism in terms of proteins composition and nutrients availability that allows the bacterium to adapt to changing environments. The presence of two cytosolic membrane-associated and two periplasmic hydrogenases in these samples pointed toward energy conservation mechanism through hydrogen-cycling under sulfate respiration (Keller and Wall, 2011; Smith et al., 2019).

Besides, the iDvu71 model was used to assess the impact of sulfate presence on hydrogen metabolism and *Dv*H physiology. In the absence of sulfate, electrons resulting from lactate oxidation led to hydrogen production (Voordouw, 1995; Noguera et al., 1998). However, in the presence of sulfate, a portion of the produced hydrogen is consumed by HysAB that provides two electrons per molecule of hydrogen for sulfate respiration (Pohorelic, 2001). As sulfate consumption increases, the demand for electrons also increases, resulting in a decrease of excreted hydrogen. Interestingly, when the sulfate/lactate flux ratio reaches 0.45, all produced hydrogen is used for sulfate respiration, and no more hydrogen is excreted by *Dv*H. This mechanism was reinforced by the experimental data, since transient hydrogen in the gas phase was also detected throughout growth (Figure 2). The above observations could be explained by the role of sulfate respiration in ATP production. For each mmole of sulfate consumed by *Dv*H, 2 mmoles of ATP are produced through a process involving the creation of a proton gradient (Pohorelic, 2001). The presence of sulfate triggers an increase in ATP production, leading to a rewiring of carbon fluxes toward gluconeogenesis and consequently to an increase in biomass synthesis which was previously determined (Noguera et al., 1998). In the absence of sulfate in the medium, the synthesis and excretion of succinate was also simulated by iDvu71. This is in agreement with previous data showing succinate excretion by *Dv*H when fumarate was used as the sole source of carbon and electrons in the absence of sulfate. Under this growth condition, succinate acted as the final electron acceptor (Zaunmüller et al., 2006).

Interestingly, the enzymes involved in formate metabolism as PFLs and FDHs isoforms were also unambiguously identified in these samples by proteomic analysis (Table 1). However, in this case, no transient formate was detected by HPLC in the cell culture supernatant. The fact that extracellular formate is not detected under lactate/sulfate growth does not mean that the pathway is not functional. Instead, the absence of formate in the cell supernatant could be explained by the fact that the concentration of excreted formate is below the HPLC detection limit. Furthermore, in light of the formate-cycling model, formate could be further metabolized at significant rate immediately after being produced. This is in line with a study from Da Silva et al. (2013) reporting that traces of formate were transiently detected during lactate/sulfate growth based on enzymatic measurements. Hence, iDvu71 was also used to give clues toward the existence of formate-cycling in the cell under sulfate respiration. Through unconstrained computational simulations, it was observed that the pathway incorporating the ferredoxin-oxidoreductase reaction is exclusively utilized (Vita et al., 2015). This contrasted with previous mutagenesis studies showing that *Dv*H growth is impaired by the inactivation of FDHs (Da Silva et al., 2013). To challenge the role of formate metabolism by constraining iDvu71 to use PFLs here we showed that *Dv*H can use indifferently PFOR or PFLs for the conversion of pyruvate into acetyl-coA with no impact on the biomass growth rate. However, the hydrogen production was affected by the replacement of PFOR activity toward PFLs activities. This could be explained by the fact that both pathways operate concurrently with electrons being transferred from hydrogen synthesis toward formate synthesis. Moreover, in sulfate-consuming conditions, formate is oxidized to carbon dioxide, releasing electrons that are then employed in sulfate respiration via cytochromes. Ultimately, further studies will be required to experimentally capture formate synthesis in *Dv*H during anaerobic sulfate respiration. Nevertheless, all these simulations provided further evidence for possible formate cycling in *Dv*H. Furthermore, while different electron donor pathways do not influence *Dv*H growth rate under lactate/sulfate respiration, it plays a key role determining the hydrogen/formate balance in the environment in the absence of sulfate. Indeed, hydrogen and formate are considered to be the primary shuttle compounds for interspecies electron transfer in anaerobic syntrophic consortia (McInerney et al., 2008; Stams and Plugge, 2009; Schink and Stams, 2013). Interestingly, syntrophic consortia can be mediated by the exclusive use of hydrogen as an electron donnor (Thiele and Zeikus, 1988; Müller et al., 2010; Teng et al., 2019), or by a combination of formate/hydrogen electron carriers (Stams and Plugge, 2009; Sieber et al., 2014; Krumholz et al., 2015). Hence, by controlling the hydrogen/formate balance in the environment, *Dv*H might be a key determinant influencing the composition of the ecosystem.

Strikingly, six Adhs, one Aor, and all the subunits forming the Hdr-Flx complex were also identified with high confidence by proteomic analysis, with Adh1 being one of the most abundant protein in these samples (Table 1). These results suggest that ethanol might act as metabolic intermediates under these conditions as previously reported (Haveman et al., 2003). Indeed, it was proposed that ethanol could serve as alternative reducing equivalents for sulfate reduction (Haveman et al., 2003; Ramos et al., 2015), through alternative energy conserving (flavin-based electron bifurcation) process involving the Hdr-Flx complex (Ramos et al., 2015). The exact mechanism of ethanol metabolism remains unclear as of today, nevertheless several scenarios were previously proposed depending on nutrients availability. Under sulfate respiration using various electron donors, ethanol is oxidized by an Adh, most likely Adh1, coupled to the reduction of NAD^+^ to NADH. The two electrons generated from NADH reoxidation by the Flx complex are then transferred through the Hdr complex to the ferredoxin pool and an unknow electron acceptor (Pereira et al., 2008; Ramos et al., 2015). It was recently proposed that DsrC might serve as electron acceptor, thus providing electrons required for sulfate reduction, while reduced ferredoxin will serve as donor for hydrogen production by membrane-associated hydrogenases (Ech or Coo) for hydrogenase production and the generation of protons gradient (Ferreira et al., 2023). Alternatively, during fermentation, the complex might work in reverse thus dissipating the excess of reducing equivalents toward ethanol production with NADH. The NAD^+^ produced is recycled by Flx complex with electrons coming from reduced ferredoxin and DsrC through Hdr complex (Ramos et al., 2015; Ferreira et al., 2023). Interestingly, according to the present study no intracellular flux for ethanol pathway was simulated using the iDvu71 model. This result suggested that the absence of ethanol as intermediate metabolite must not prevent growth nor impact hydrogen and formate production in *Dv*H under sulfate respiration.

To conclude, coupling fluxomic and proteomic analysis, the present study provided a comprehensive understanding of *Dv*H primary metabolism intracellular fluxes. The contribution of different energy conservation mechanisms involving the cycling of intermediates on growth and hydrogen production was evaluated depending on the presence of sulfate as electron acceptor. Altogether, this study emphasized that the flexibility of *Dv*H metabolic capabilities most likely lies in environmental conditions which determine enzymes composition in *Dv*H, rather than bioenergetic advantages. Besides, this study also highlighted the importance of coupling fluxomic to experimental data to simulate accurate intracellular fluxes. Moreover, besides enabling *Dv*H to grow and persist in various ecosystems, this breadth of metabolic capability most likely confers to *Dv*H a central role in shaping microbiome composition and function. Developing models relevant in a variety of environmental conditions remains challenging. Most of the times, environmental changes require adjustments in the metabolic network to obtain accurate simulations. Comparison of iDvu71 results with those obtained with previously developed metabolic models of *Dv*H illustrated this difficulty. To date, iDvu71 was only used to simulate the metabolic behavior of *Dv*H with constrained lactate and sulfate consumption fluxes. However, this model will be part of a metabolic model for an artificial community composed of *C. acetobutylicum* and *Dv*H. An overproduction of hydrogen was determined in this consortium compared to a *C. acetobutylicum* pure culture (Benomar et al., 2015; Barca et al., 2016). This community metabolic model, including iDvu71, should provide insights regarding the metabolite exchanges between these two bacteria which could be the basis of this overproduction.

## Data availability statement

The data presented in the study are deposited in the ProteomeXchange Consortium via the PRIDE partner repository (http://www.ebi.ac.uk/pride) with the ProteomeXchange, accession PXD046638.

## Author contributions

XM: Writing – original draft, Conceptualization, Data curation, Formal Analysis, Investigation, Methodology, Software, Validation, Visualization. MR: Conceptualization, Data curation, Formal Analysis, Investigation, Methodology, Validation, Visualization, Writing – original draft, Writing – review and editing, Supervision. FF: Conceptualization, Formal Analysis, Methodology, Software, Supervision, Writing – review and editing. PI: Conceptualization, Data curation, Formal Analysis, Investigation, Methodology, Writing – review and editing, Validation. EG: Conceptualization, Investigation, Supervision, Writing – review and editing. LD: Data curation, Formal Analysis, Investigation, Methodology, Writing – review and editing. RL: Data curation, Formal Analysis, Investigation, Methodology, Resources, Validation, Writing – review and editing. M-TG-O: Conceptualization, Funding acquisition, Project administration, Supervision, Validation, Writing – review and editing, Data curation, Formal Analysis. SD: Conceptualization, Data curation, Formal Analysis, Funding acquisition, Investigation, Methodology, Supervision, Visualization, Writing – review and editing.
