# Supplementary material for: Combining metabolic flux analysis with proteomics to shed light on the metabolic flexibility: the case of Desulfovibrio vulgaris Hildenborough
Source: Front Microbiol. 2024 Feb 23;15:1336360. doi: 10.3389/fmicb.2024.1336360 (PMC10920352; doi:10.3389/fmicb.2024.1336360)
Supplement: Supplementary file 1 [file Data_Sheet_1.PDF]

```
<?xml version="1.0" encoding="UTF-8"?>
<sbml xmlns="http://www.sbml.org/sbml/level2" level="2" version="1"
xmlns:html="http://www.w3.org/1999/xhtml">
  <model id="Dvulgaris" name="Desulfovibrio vulgaris">
  <listOfUnitDefinitions>
      <unitDefinition id="mmol_per_gDW_per_hr">
        <listOfUnits>
          <unit kind="mole" scale="-3" multiplier="1" offset="0" />
          <unit kind="gram" exponent="-1" multiplier="1" offset="0" />
          <unit kind="second" exponent="-1"
multiplier="0.00027777777777778" offset="0" />
        </listOfUnits>
      </unitDefinition>
    </listOfUnitDefinitions>
    <listOfCompartments>
      <compartment id="c" name="Cytoplasm"/>
      <compartment id="e" name="Extracellular"/>
    </listOfCompartments>
    <listOfSpecies>
      <species compartment="c" id="M_13bpg_c" name="1,3-Bisphospho-D-
glycerate"/>
      <species compartment="c" id="M_2pg_c" name="2-phospho-D-glycerate"/>
      <species compartment="c" id="M_3pg_c" name="3-phospho-D-glycerate"/>
      <species compartment="c" id="M_ac_c" name="Acetate"/>
      <species compartment="c" id="M_accoa_c" name="Acetyl-CoA"/>
      <species compartment="c" id="M_actp_c" name="Acetyl Phosphate"/>
      <species compartment="c" id="M_adp_c" name="Adenosine 5'-
diphosphate"/>
      <species compartment="c" id="M_amp_c" name="Adenosine 5'-phosphate"/>
      <species compartment="c" id="M_aps_c" name="Adenylyl sulfate"/>
      <species compartment="c" id="M_atp_c" name="Adenosine 5'-
triphosphate"/>
      <species compartment="c" id="M_cit_c" name="Citrate"/>
      <species compartment="c" id="M_co2_c" name="Dioxyde Carbon"/>
      <species compartment="c" id="M_coa_c" name="CoA"/>
      <species compartment="c" id="M_dhap_c" name="Dihydroxyacétone
phosphate"/>
      <species compartment="c" id="M_e4p_c" name="D-Erythrose 4-
phosphate"/>
      <species compartment="c" id="M_f6p_c" name="D-fructose-6-phosphate"/>
      <species compartment="c" id="M_fbp_c" name="D-Fructose 1,6-
bisphosphate"/>
      <species compartment="c" id="M_fedox_c" name="federoxine oxyde"/>
      <species compartment="c" id="M_fedred_c" name="Federoxine reduite"/>
      <species compartment="c" id="M_fum_c" name="fumarate"/>
      <species compartment="c" id="M_g3p_c" name="Glyceraldehyde-3-
phosphate"/>
      <species compartment="c" id="M_g6p_c" name="Glucose 6-phosphate"/>
      <species compartment="c" id="M_h_c" name="Proton"/>
      <species compartment="c" id="M_h2_c" name="Hydrogen"/>
      <species compartment="c" id="M_h2o_c" name="Water"/>
      <species compartment="c" id="M_h2s_c" name="Hydrogen Sulfide"/>
      <species compartment="c" id="M_icit_c" name="Isocitrate"/>
      <species compartment="c" id="M_lac_c" name="Lactate"/>
      <species compartment="c" id="M_mal_c" name="malate"/>
      <species compartment="c" id="M_nad_c" name="NAD"/>
      <species compartment="c" id="M_nadh_c" name="NADH"/>
      <species compartment="c" id="M_oaa_c" name="Oxaloacetate"/>
      <species compartment="c" id="M_oxo_c" name="Oxoglutarate"/>
      <species compartment="c" id="M_pep_c" name="Phosphoenolpyruvate"/>
      <species compartment="c" id="M_pi_c" name="Phosphate inorganique"/>
```

```
      <species compartment="c" id="M_ppi_c" name="Biphosphate"/>
      <species compartment="c" id="M_pyr_c" name="Pyruvate"/>
      <species compartment="c" id="M_quinol_c" name="quinol"/>
      <species compartment="c" id="M_quinone_c" name="quinone"/>
      <species compartment="c" id="M_ribo5p_c" name="Ribose-5-phophate"/>
      <species compartment="c" id="M_ribu5p_c" name="Ribulose-5-
phosphate"/>
      <species compartment="c" id="M_s7p_c" name="Sedoheptulose-7-
phosphate"/>
      <species compartment="c" id="M_so3_c" name="Sulfite"/>
      <species compartment="c" id="M_succ_c" name="Succinate"/>
      <species compartment="c" id="M_succoa_c" name="Succinyl-Coa"/>
      <species compartment="c" id="M_sulfate_c" name="sulfate"/>
      <species compartment="c" id="M_xyl5p_c" name="Xylose-5-posphate"/>
      <species compartment="c" id="M_form_c" name="Formate"/>
      <species compartment="c" id="M_etoh_c" name="Ethanol"/>
      <species compartment="c" id="M_acald_c" name="acetaldehyde"/>
      <species compartment="c" id="M_biomasse_c" name="Biomasse"/>
      <species compartment="e" id="M_co2_e" name="Dioxyde Carbon"/>
      <species compartment="e" id="M_ac_e" name="Acetate"/>
      <species compartment="e" id="M_h_e" name="Proton"/>
      <species compartment="e" id="M_h2_e" name="Hydrogen"/>
      <species compartment="e" id="M_h2o_e" name="Water"/>
      <species compartment="e" id="M_h2s_e" name="Hydrogen Sulfide"/>
      <species compartment="e" id="M_lac_e" name="Lactate"/>
      <species compartment="e" id="M_pi_e" name="Phosphate inorganique"/>
      <species compartment="e" id="M_sulfate_e" name="sulfate"/>
      <species compartment="e" id="M_form_e" name="Formate"/>
      <species compartment="e" id="M_etoh_e" name="Ethanol"/>
      <species compartment="e" id="M_biomasse_e" name="Biomasse"/>
      <species compartment="e" id="M_c3ox_e" name="cytochrome oxyde"/>
      <species compartment="e" id="M_c3red_e" name="Cytochrome reduit"/>
      <species compartment="e" id="M_succ_e" name="Succinate"/>
      <species compartment="e" id="M_adp_e" name="Adenosine 5'-
diphosphate"/>
    </listOfSpecies>
    <listOfReactions>
      <reaction id="R_lactate_EX" name="1 lac[e] = " reversible="1">
        <notes>
          <html:p>GENE_ASSOCIATION:  </html:p>
          <html:p>SUBSYSTEM:  base</html:p>
          <html:p>note: </html:p>
          <html:p>Equation: 1 lac[e] =</html:p>
        </notes>
        <listOfReactants>
          <speciesReference species="M_lac_e" stoichiometry="1"/>
        </listOfReactants>
        <kineticLaw>
          <math xmlns="http://www.w3.org/1998/Math/MathML">
            <ci> FLUX_VALUE </ci>
          </math>
          <listOfParameters>
            <parameter id="LOWER_BOUND" value="-100"
units="mmol_per_gDW_per_hr"/>
            <parameter id="UPPER_BOUND" value="100"
units="mmol_per_gDW_per_hr"/>
            <parameter id="FLUX_VALUE" value="0"
units="mmol_per_gDW_per_hr"/>
            <parameter id="OBJECTIVE_COEFFICIENT" value="0"
units="mmol_per_gDW_per_hr"/>
          </listOfParameters>
```

```
        </kineticLaw>
      </reaction>
      <reaction id="R_phosphate_EX" name="1 pi[e] = " reversible="1">
        <notes>
          <html:p>GENE_ASSOCIATION:  </html:p>
          <html:p>SUBSYSTEM:  base</html:p>
          <html:p>note: </html:p>
          <html:p>Equation: 1 pi[e] =</html:p>
        </notes>
        <listOfReactants>
          <speciesReference species="M_pi_e" stoichiometry="1"/>
        </listOfReactants>
        <kineticLaw>
          <math xmlns="http://www.w3.org/1998/Math/MathML">
            <ci> FLUX_VALUE </ci>
          </math>
          <listOfParameters>
            <parameter id="LOWER_BOUND" value="-100"
units="mmol_per_gDW_per_hr"/>
            <parameter id="UPPER_BOUND" value="100"
units="mmol_per_gDW_per_hr"/>
            <parameter id="FLUX_VALUE" value="0"
units="mmol_per_gDW_per_hr"/>
            <parameter id="OBJECTIVE_COEFFICIENT" value="0"
units="mmol_per_gDW_per_hr"/>
          </listOfParameters>
        </kineticLaw>
      </reaction>
      <reaction id="R_ethanol_EX" name="1 etoh[e] = " reversible="1">
        <notes>
          <html:p>GENE_ASSOCIATION:  </html:p>
          <html:p>SUBSYSTEM:  base</html:p>
          <html:p>note: </html:p>
          <html:p>Equation: 1 etoh[e] = 1 etoh[b]</html:p>
        </notes>
        <listOfReactants>
          <speciesReference species="M_etoh_e" stoichiometry="1"/>
        </listOfReactants>
        <kineticLaw>
          <math xmlns="http://www.w3.org/1998/Math/MathML">
            <ci> FLUX_VALUE </ci>
          </math>
          <listOfParameters>
            <parameter id="LOWER_BOUND" value="-100"
units="mmol_per_gDW_per_hr"/>
            <parameter id="UPPER_BOUND" value="100"
units="mmol_per_gDW_per_hr"/>
            <parameter id="FLUX_VALUE" value="0"
units="mmol_per_gDW_per_hr"/>
            <parameter id="OBJECTIVE_COEFFICIENT" value="0"
units="mmol_per_gDW_per_hr"/>
          </listOfParameters>
        </kineticLaw>
      </reaction>
      <reaction id="R_acetate_EX" name="1 ac[e] = " reversible="1">
        <notes>
          <html:p>GENE_ASSOCIATION:  </html:p>
          <html:p>SUBSYSTEM:  base</html:p>
          <html:p>note: </html:p>
          <html:p>Equation: 1 ac[e] =</html:p>
        </notes>
```

```
        <listOfReactants>
          <speciesReference species="M_ac_e" stoichiometry="1"/>
        </listOfReactants>
        <kineticLaw>
          <math xmlns="http://www.w3.org/1998/Math/MathML">
            <ci> FLUX_VALUE </ci>
          </math>
          <listOfParameters>
            <parameter id="LOWER_BOUND" value="-100"
units="mmol_per_gDW_per_hr"/>
            <parameter id="UPPER_BOUND" value="100"
units="mmol_per_gDW_per_hr"/>
            <parameter id="FLUX_VALUE" value="0"
units="mmol_per_gDW_per_hr"/>
            <parameter id="OBJECTIVE_COEFFICIENT" value="0"
units="mmol_per_gDW_per_hr"/>
          </listOfParameters>
        </kineticLaw>
      </reaction>
      <reaction id="R_sulfate_EX" name="1 sulfate[e] = " reversible="1">
        <notes>
          <html:p>GENE_ASSOCIATION:  </html:p>
          <html:p>SUBSYSTEM:  base</html:p>
          <html:p>note: </html:p>
          <html:p>Equation: 1 sulfate[e] =</html:p>
        </notes>
        <listOfReactants>
          <speciesReference species="M_sulfate_e" stoichiometry="1"/>
        </listOfReactants>
        <kineticLaw>
          <math xmlns="http://www.w3.org/1998/Math/MathML">
            <ci> FLUX_VALUE </ci>
          </math>
          <listOfParameters>
            <parameter id="LOWER_BOUND" value="-100"
units="mmol_per_gDW_per_hr"/>
            <parameter id="UPPER_BOUND" value="100"
units="mmol_per_gDW_per_hr"/>
            <parameter id="FLUX_VALUE" value="0"
units="mmol_per_gDW_per_hr"/>
            <parameter id="OBJECTIVE_COEFFICIENT" value="0"
units="mmol_per_gDW_per_hr"/>
          </listOfParameters>
        </kineticLaw>
      </reaction>
      <reaction id="R_hydrogen_sulfide_EX" name="1 h2s[e] = "
reversible="1">
        <notes>
          <html:p>GENE_ASSOCIATION:  </html:p>
          <html:p>SUBSYSTEM:  base</html:p>
          <html:p>note: </html:p>
          <html:p>Equation: 1 h2s[e] =</html:p>
        </notes>
        <listOfReactants>
          <speciesReference species="M_h2s_e" stoichiometry="1"/>
        </listOfReactants>
        <kineticLaw>
          <math xmlns="http://www.w3.org/1998/Math/MathML">
            <ci> FLUX_VALUE </ci>
          </math>
          <listOfParameters>
```

```
            <parameter id="LOWER_BOUND" value="-100"
units="mmol_per_gDW_per_hr"/>
            <parameter id="UPPER_BOUND" value="100"
units="mmol_per_gDW_per_hr"/>
            <parameter id="FLUX_VALUE" value="0"
units="mmol_per_gDW_per_hr"/>
            <parameter id="OBJECTIVE_COEFFICIENT" value="0"
units="mmol_per_gDW_per_hr"/>
          </listOfParameters>
        </kineticLaw>
      </reaction>
      <reaction id="R_formate_EX" name="1 form[e] = " reversible="1">
        <notes>
          <html:p>GENE_ASSOCIATION:  </html:p>
          <html:p>SUBSYSTEM:  base</html:p>
          <html:p>note: </html:p>
          <html:p>Equation: 1 form[e] =</html:p>
        </notes>
        <listOfReactants>
          <speciesReference species="M_form_e" stoichiometry="1"/>
        </listOfReactants>
        <kineticLaw>
          <math xmlns="http://www.w3.org/1998/Math/MathML">
            <ci> FLUX_VALUE </ci>
          </math>
          <listOfParameters>
            <parameter id="LOWER_BOUND" value="-100"
units="mmol_per_gDW_per_hr"/>
            <parameter id="UPPER_BOUND" value="100"
units="mmol_per_gDW_per_hr"/>
            <parameter id="FLUX_VALUE" value="0"
units="mmol_per_gDW_per_hr"/>
            <parameter id="OBJECTIVE_COEFFICIENT" value="0"
units="mmol_per_gDW_per_hr"/>
          </listOfParameters>
        </kineticLaw>
      </reaction>
      <reaction id="R_Co2_EX" name="1 co2[e] = " reversible="1">
        <notes>
          <html:p>GENE_ASSOCIATION:  </html:p>
          <html:p>SUBSYSTEM:  base</html:p>
          <html:p>note: </html:p>
          <html:p>Equation: 1 co2[e] =</html:p>
        </notes>
        <listOfReactants>
          <speciesReference species="M_co2_e" stoichiometry="1"/>
        </listOfReactants>
        <kineticLaw>
          <math xmlns="http://www.w3.org/1998/Math/MathML">
            <ci> FLUX_VALUE </ci>
          </math>
          <listOfParameters>
            <parameter id="LOWER_BOUND" value="-100"
units="mmol_per_gDW_per_hr"/>
            <parameter id="UPPER_BOUND" value="100"
units="mmol_per_gDW_per_hr"/>
            <parameter id="FLUX_VALUE" value="0"
units="mmol_per_gDW_per_hr"/>
            <parameter id="OBJECTIVE_COEFFICIENT" value="0"
units="mmol_per_gDW_per_hr"/>
          </listOfParameters>
```

```
        </kineticLaw>
      </reaction>
      <reaction id="R_proton_EX" name="1 h[e] = " reversible="1">
        <notes>
          <html:p>GENE_ASSOCIATION:  </html:p>
          <html:p>SUBSYSTEM:  base</html:p>
          <html:p>note: </html:p>
          <html:p>Equation: 1 h[e] =</html:p>
        </notes>
        <listOfReactants>
          <speciesReference species="M_h_e" stoichiometry="1"/>
        </listOfReactants>
        <kineticLaw>
          <math xmlns="http://www.w3.org/1998/Math/MathML">
            <ci> FLUX_VALUE </ci>
          </math>
          <listOfParameters>
            <parameter id="LOWER_BOUND" value="-100"
units="mmol_per_gDW_per_hr"/>
            <parameter id="UPPER_BOUND" value="100"
units="mmol_per_gDW_per_hr"/>
            <parameter id="FLUX_VALUE" value="0"
units="mmol_per_gDW_per_hr"/>
            <parameter id="OBJECTIVE_COEFFICIENT" value="0"
units="mmol_per_gDW_per_hr"/>
          </listOfParameters>
        </kineticLaw>
      </reaction>
      <reaction id="R_succinate_EX" name="1 succ[e] = " reversible="1">
        <notes>
          <html:p>GENE_ASSOCIATION:  </html:p>
          <html:p>SUBSYSTEM:  base</html:p>
          <html:p>note: </html:p>
          <html:p>Equation: 1 succ[e] =</html:p>
        </notes>
        <listOfReactants>
          <speciesReference species="M_succ_e" stoichiometry="1"/>
        </listOfReactants>
        <kineticLaw>
          <math xmlns="http://www.w3.org/1998/Math/MathML">
            <ci> FLUX_VALUE </ci>
          </math>
          <listOfParameters>
            <parameter id="LOWER_BOUND" value="-100"
units="mmol_per_gDW_per_hr"/>
            <parameter id="UPPER_BOUND" value="100"
units="mmol_per_gDW_per_hr"/>
            <parameter id="FLUX_VALUE" value="0"
units="mmol_per_gDW_per_hr"/>
            <parameter id="OBJECTIVE_COEFFICIENT" value="0"
units="mmol_per_gDW_per_hr"/>
          </listOfParameters>
        </kineticLaw>
      </reaction>
      <reaction id="R_hydrogen_EX" name="1 h2[e] = " reversible="1">
        <notes>
          <html:p>GENE_ASSOCIATION:  </html:p>
          <html:p>SUBSYSTEM:  base</html:p>
          <html:p>note: </html:p>
          <html:p>Equation: 1 h2[e] =</html:p>
        </notes>
```

```
        <listOfReactants>
          <speciesReference species="M_h2_e" stoichiometry="1"/>
        </listOfReactants>
        <kineticLaw>
          <math xmlns="http://www.w3.org/1998/Math/MathML">
            <ci> FLUX_VALUE </ci>
          </math>
          <listOfParameters>
            <parameter id="LOWER_BOUND" value="-100"
units="mmol_per_gDW_per_hr"/>
            <parameter id="UPPER_BOUND" value="100"
units="mmol_per_gDW_per_hr"/>
            <parameter id="FLUX_VALUE" value="0"
units="mmol_per_gDW_per_hr"/>
            <parameter id="OBJECTIVE_COEFFICIENT" value="0"
units="mmol_per_gDW_per_hr"/>
          </listOfParameters>
        </kineticLaw>
      </reaction>
      <reaction id="R_Water_Ex" name="1 h2o[e] = " reversible="1">
        <notes>
          <html:p>GENE_ASSOCIATION:  </html:p>
          <html:p>SUBSYSTEM:  base</html:p>
          <html:p>note: </html:p>
          <html:p>Equation: 1 h2o[e] =</html:p>
        </notes>
        <listOfReactants>
          <speciesReference species="M_h2o_e" stoichiometry="1"/>
        </listOfReactants>
        <kineticLaw>
          <math xmlns="http://www.w3.org/1998/Math/MathML">
            <ci> FLUX_VALUE </ci>
          </math>
          <listOfParameters>
            <parameter id="LOWER_BOUND" value="-100"
units="mmol_per_gDW_per_hr"/>
            <parameter id="UPPER_BOUND" value="100"
units="mmol_per_gDW_per_hr"/>
            <parameter id="FLUX_VALUE" value="0"
units="mmol_per_gDW_per_hr"/>
            <parameter id="OBJECTIVE_COEFFICIENT" value="0"
units="mmol_per_gDW_per_hr"/>
          </listOfParameters>
        </kineticLaw>
      </reaction>
      <reaction id="R_bioimasse_EX" name="1 biomasse[e] = " reversible="1">
        <notes>
          <html:p>GENE_ASSOCIATION:  </html:p>
          <html:p>SUBSYSTEM:  base</html:p>
          <html:p>note: </html:p>
          <html:p>Equation: 1 biomasse[e] =</html:p>
        </notes>
        <listOfReactants>
          <speciesReference species="M_biomasse_e" stoichiometry="1"/>
        </listOfReactants>
        <kineticLaw>
          <math xmlns="http://www.w3.org/1998/Math/MathML">
            <ci> FLUX_VALUE </ci>
          </math>
          <listOfParameters>
```

```
            <parameter id="LOWER_BOUND" value="-100"
units="mmol_per_gDW_per_hr"/>
            <parameter id="UPPER_BOUND" value="100"
units="mmol_per_gDW_per_hr"/>
            <parameter id="FLUX_VALUE" value="0"
units="mmol_per_gDW_per_hr"/>
            <parameter id="OBJECTIVE_COEFFICIENT" value="0"
units="mmol_per_gDW_per_hr"/>
          </listOfParameters>
        </kineticLaw>
      </reaction>
      <reaction id="R_Biomasse" name="0,966 g6p[c] + 0,69 xyl5p[c] + 0,276
e4p[c] + 1,242 3pg[c] + 0,552 pep[c] + 3,45 pyr[c] + 2,208 accoa[c] + 1,518
oaa[c] + 0,966 oxo[c] + 96 atp[c] --> 1 biomasse[c] + 96 adp[c] + 96 pi[c]
+ 2,208 coa[c]" reversible="0">
        <notes>
          <html:p>GENE_ASSOCIATION:  </html:p>
          <html:p>SUBSYSTEM:  Biomasse equation</html:p>
          <html:p>note: </html:p>
          <html:p>Equation: 0,966 g6p[c] + 0,69 xyl5p[c] + 0,276 e4p[c] +
1,242 3pg[c] + 0,552 pep[c] + 3,45 pyr[c] + 2,208 accoa[c] + 1,518 oaa[c] +
0,966 oxo[c] + 96 atp[c] --> 1 biomasse[c] + 96 adp[c] + 96 pi[c] + 2,208
coa[c]</html:p>
        </notes>
        <listOfReactants>
          <speciesReference species="M_g6p_c" stoichiometry="0.966"/>
          <speciesReference species="M_xyl5p_c" stoichiometry="0.69"/>
          <speciesReference species="M_e4p_c" stoichiometry="0.276"/>
          <speciesReference species="M_3pg_c" stoichiometry="1.242"/>
          <speciesReference species="M_pep_c" stoichiometry="0.552"/>
          <speciesReference species="M_pyr_c" stoichiometry="3.45"/>
          <speciesReference species="M_accoa_c" stoichiometry="2.208"/>
          <speciesReference species="M_oaa_c" stoichiometry="1.518"/>
          <speciesReference species="M_oxo_c" stoichiometry="0.966"/>
          <speciesReference species="M_atp_c" stoichiometry="96"/>
        </listOfReactants>
        <listOfProducts>
          <speciesReference species="M_biomasse_c" stoichiometry="1"/>
          <speciesReference species="M_adp_c" stoichiometry="96"/>
          <speciesReference species="M_pi_c" stoichiometry="96"/>
          <speciesReference species="M_coa_c" stoichiometry="2.208"/>
        </listOfProducts>
        <kineticLaw>
          <math xmlns="http://www.w3.org/1998/Math/MathML">
            <ci> FLUX_VALUE </ci>
          </math>
          <listOfParameters>
            <parameter id="LOWER_BOUND" value="-100"
units="mmol_per_gDW_per_hr"/>
            <parameter id="UPPER_BOUND" value="100"
units="mmol_per_gDW_per_hr"/>
            <parameter id="FLUX_VALUE" value="0"
units="mmol_per_gDW_per_hr"/>
            <parameter id="OBJECTIVE_COEFFICIENT" value="1"
units="mmol_per_gDW_per_hr"/>
          </listOfParameters>
        </kineticLaw>
      </reaction>
      <reaction id="R_Alcool_deshydrogenase" name="1 etoh[c] + 1 nad[c] -->
1 acald[c] + 1 nadh[c]" reversible="0">
        <notes>
```

```
          <html:p>GENE_ASSOCIATION:  DVU2396</html:p>
          <html:p>SUBSYSTEM:  dissimilation ethanol</html:p>
          <html:p>note: </html:p>
          <html:p>Equation: 1 etoh[c] + 1 nad[c] --> 1 acald[c] + 1
nadh[c]</html:p>
        </notes>
        <listOfReactants>
          <speciesReference species="M_etoh_c" stoichiometry="1"/>
          <speciesReference species="M_nad_c" stoichiometry="1"/>
        </listOfReactants>
        <listOfProducts>
          <speciesReference species="M_acald_c" stoichiometry="1"/>
          <speciesReference species="M_nadh_c" stoichiometry="1"/>
        </listOfProducts>
        <kineticLaw>
          <math xmlns="http://www.w3.org/1998/Math/MathML">
            <ci> FLUX_VALUE </ci>
          </math>
          <listOfParameters>
            <parameter id="LOWER_BOUND" value="0"
units="mmol_per_gDW_per_hr"/>
            <parameter id="UPPER_BOUND" value="100"
units="mmol_per_gDW_per_hr"/>
            <parameter id="FLUX_VALUE" value="0"
units="mmol_per_gDW_per_hr"/>
            <parameter id="OBJECTIVE_COEFFICIENT" value="0"
units="mmol_per_gDW_per_hr"/>
          </listOfParameters>
        </kineticLaw>
      </reaction>
      <reaction id="R_Aldehyde_dehydrogenase" name="1 acald[c] + 1 nad[c] -
-> 1 ac[c] + 1 nadh[c]" reversible="0">
        <notes>
          <html:p>GENE_ASSOCIATION:  </html:p>
          <html:p>SUBSYSTEM:  dissimilation ethanol</html:p>
          <html:p>note: </html:p>
          <html:p>Equation: 1 acald[c] + 1 nad[c] --> 1 ac[c] + 1
nadh[c]</html:p>
        </notes>
        <listOfReactants>
          <speciesReference species="M_acald_c" stoichiometry="1"/>
          <speciesReference species="M_nad_c" stoichiometry="1"/>
        </listOfReactants>
        <listOfProducts>
          <speciesReference species="M_ac_c" stoichiometry="1"/>
          <speciesReference species="M_nadh_c" stoichiometry="1"/>
        </listOfProducts>
        <kineticLaw>
          <math xmlns="http://www.w3.org/1998/Math/MathML">
            <ci> FLUX_VALUE </ci>
          </math>
          <listOfParameters>
            <parameter id="LOWER_BOUND" value="0"
units="mmol_per_gDW_per_hr"/>
            <parameter id="UPPER_BOUND" value="100"
units="mmol_per_gDW_per_hr"/>
            <parameter id="FLUX_VALUE" value="0"
units="mmol_per_gDW_per_hr"/>
            <parameter id="OBJECTIVE_COEFFICIENT" value="0"
units="mmol_per_gDW_per_hr"/>
          </listOfParameters>
```

```
        </kineticLaw>
      </reaction>
      <reaction id="R_sulfate_adenylyltransferase" name="1 sulfate[c] + 1
atp[c] = 1 ppi[c] + 1 aps[c]" reversible="1">
        <notes>
          <html:p>GENE_ASSOCIATION:  DVU1295</html:p>
          <html:p>SUBSYSTEM:  Dissimilation_sulfate</html:p>
          <html:p>note: </html:p>
          <html:p>Equation: 1 sulfate[c] + 1 atp[c] = 1 ppi[c] + 1
aps[c]</html:p>
        </notes>
        <listOfReactants>
          <speciesReference species="M_sulfate_c" stoichiometry="1"/>
          <speciesReference species="M_atp_c" stoichiometry="1"/>
        </listOfReactants>
        <listOfProducts>
          <speciesReference species="M_ppi_c" stoichiometry="1"/>
          <speciesReference species="M_aps_c" stoichiometry="1"/>
        </listOfProducts>
        <kineticLaw>
          <math xmlns="http://www.w3.org/1998/Math/MathML">
            <ci> FLUX_VALUE </ci>
          </math>
          <listOfParameters>
            <parameter id="LOWER_BOUND" value="-100"
units="mmol_per_gDW_per_hr"/>
            <parameter id="UPPER_BOUND" value="100"
units="mmol_per_gDW_per_hr"/>
            <parameter id="FLUX_VALUE" value="0"
units="mmol_per_gDW_per_hr"/>
            <parameter id="OBJECTIVE_COEFFICIENT" value="0"
units="mmol_per_gDW_per_hr"/>
          </listOfParameters>
        </kineticLaw>
      </reaction>
      <reaction id="R_adenylylsulfate_reductase" name="1 aps[c] + 1
c3red[e] + 2 h[c] --> 1 so3[c] + 1 c3ox[e] + 1 amp[c]" reversible="1">
        <notes>
          <html:p>GENE_ASSOCIATION:  DVU0846</html:p>
          <html:p>SUBSYSTEM:  Dissimilation_sulfate</html:p>
          <html:p>note: </html:p>
          <html:p>Equation: 1 aps[c] + 1 c3red[e] + 2 h[c] --> 1 so3[c] + 1
c3ox[e] + 1 amp[c]</html:p>
        </notes>
        <listOfReactants>
          <speciesReference species="M_aps_c" stoichiometry="1"/>
          <speciesReference species="M_c3red_e" stoichiometry="1"/>
          <speciesReference species="M_h_c" stoichiometry="2"/>
        </listOfReactants>
        <listOfProducts>
          <speciesReference species="M_so3_c" stoichiometry="1"/>
          <speciesReference species="M_c3ox_e" stoichiometry="1"/>
          <speciesReference species="M_amp_c" stoichiometry="1"/>
        </listOfProducts>
        <kineticLaw>
          <math xmlns="http://www.w3.org/1998/Math/MathML">
            <ci> FLUX_VALUE </ci>
          </math>
          <listOfParameters>
            <parameter id="LOWER_BOUND" value="-100"
units="mmol_per_gDW_per_hr"/>
```

```
            <parameter id="UPPER_BOUND" value="100"
units="mmol_per_gDW_per_hr"/>
            <parameter id="FLUX_VALUE" value="0"
units="mmol_per_gDW_per_hr"/>
            <parameter id="OBJECTIVE_COEFFICIENT" value="0"
units="mmol_per_gDW_per_hr"/>
          </listOfParameters>
        </kineticLaw>
      </reaction>
      <reaction id="R_dissimilatory_sulfite_reductase" name="1 so3[c] + 6
h[c] + 3 c3red[e] --> 1 h2s[c] + 3 h2o[c] + 3 c3ox[e]" reversible="1">
        <notes>
          <html:p>GENE_ASSOCIATION:  DVU0402</html:p>
          <html:p>SUBSYSTEM:  Dissimilation_sulfate</html:p>
          <html:p>note: </html:p>
          <html:p>Equation: 1 so3[c] + 6 h[c] + 3 c3red[e] --> 1 h2s[c] + 3
h2o[c] + 3 c3ox[e]</html:p>
        </notes>
        <listOfReactants>
          <speciesReference species="M_so3_c" stoichiometry="1"/>
          <speciesReference species="M_h_c" stoichiometry="6"/>
          <speciesReference species="M_c3red_e" stoichiometry="3"/>
        </listOfReactants>
        <listOfProducts>
          <speciesReference species="M_h2s_c" stoichiometry="1"/>
          <speciesReference species="M_h2o_c" stoichiometry="3"/>
          <speciesReference species="M_c3ox_e" stoichiometry="3"/>
        </listOfProducts>
        <kineticLaw>
          <math xmlns="http://www.w3.org/1998/Math/MathML">
            <ci> FLUX_VALUE </ci>
          </math>
          <listOfParameters>
            <parameter id="LOWER_BOUND" value="0"
units="mmol_per_gDW_per_hr"/>
            <parameter id="UPPER_BOUND" value="100"
units="mmol_per_gDW_per_hr"/>
            <parameter id="FLUX_VALUE" value="0"
units="mmol_per_gDW_per_hr"/>
            <parameter id="OBJECTIVE_COEFFICIENT" value="0"
units="mmol_per_gDW_per_hr"/>
          </listOfParameters>
        </kineticLaw>
      </reaction>
      <reaction id="R_hydrogenase" name="1 fedred[c] = 1 fedox[c] + 1
h2[c]" reversible="1">
        <notes>
          <html:p>GENE_ASSOCIATION:  DVU</html:p>
          <html:p>SUBSYSTEM:  exchange_electron</html:p>
          <html:p>note: </html:p>
          <html:p>Equation: 1 fedred[c] = 1 fedox[c] + 1 h2[c]</html:p>
        </notes>
        <listOfReactants>
          <speciesReference species="M_fedred_c" stoichiometry="1"/>
        </listOfReactants>
        <listOfProducts>
          <speciesReference species="M_fedox_c" stoichiometry="1"/>
          <speciesReference species="M_h2_c" stoichiometry="1"/>
        </listOfProducts>
        <kineticLaw>
          <math xmlns="http://www.w3.org/1998/Math/MathML">
```

```
            <ci> FLUX_VALUE </ci>
          </math>
          <listOfParameters>
            <parameter id="LOWER_BOUND" value="-100"
units="mmol_per_gDW_per_hr"/>
            <parameter id="UPPER_BOUND" value="100"
units="mmol_per_gDW_per_hr"/>
            <parameter id="FLUX_VALUE" value="0"
units="mmol_per_gDW_per_hr"/>
            <parameter id="OBJECTIVE_COEFFICIENT" value="0"
units="mmol_per_gDW_per_hr"/>
          </listOfParameters>
        </kineticLaw>
      </reaction>
      <reaction id="R_fedroxine_hydrogenase" name="1 nadh[c] + 1 fedox[c] =
1 nad[c] + 1 fedred[c]" reversible="1">
        <notes>
          <html:p>GENE_ASSOCIATION:  DVU</html:p>
          <html:p>SUBSYSTEM:  exchange_electron</html:p>
          <html:p>note: </html:p>
          <html:p>Equation: 1 nadh[c] + 1 fedox[c] = 1 nad[c] + 1
fedred[c]</html:p>
        </notes>
        <listOfReactants>
          <speciesReference species="M_nadh_c" stoichiometry="1"/>
          <speciesReference species="M_fedox_c" stoichiometry="1"/>
        </listOfReactants>
        <listOfProducts>
          <speciesReference species="M_nad_c" stoichiometry="1"/>
          <speciesReference species="M_fedred_c" stoichiometry="1"/>
        </listOfProducts>
        <kineticLaw>
          <math xmlns="http://www.w3.org/1998/Math/MathML">
            <ci> FLUX_VALUE </ci>
          </math>
          <listOfParameters>
            <parameter id="LOWER_BOUND" value="-100"
units="mmol_per_gDW_per_hr"/>
            <parameter id="UPPER_BOUND" value="100"
units="mmol_per_gDW_per_hr"/>
            <parameter id="FLUX_VALUE" value="0"
units="mmol_per_gDW_per_hr"/>
            <parameter id="OBJECTIVE_COEFFICIENT" value="0"
units="mmol_per_gDW_per_hr"/>
          </listOfParameters>
        </kineticLaw>
      </reaction>
      <reaction id="R_cytochrome_reductase" name="1 c3ox[e] + 1 h2[e] --> 1
c3red[e] + 2 h[e]" reversible="1">
        <notes>
          <html:p>GENE_ASSOCIATION:  DVU</html:p>
          <html:p>SUBSYSTEM:  exchange_electron</html:p>
          <html:p>note: </html:p>
          <html:p>Equation: 1 c3ox[e] + 1 h2[e] --> 1 c3red[e] + 2
h[e]</html:p>
        </notes>
        <listOfReactants>
          <speciesReference species="M_c3ox_e" stoichiometry="1"/>
          <speciesReference species="M_h2_e" stoichiometry="1"/>
        </listOfReactants>
        <listOfProducts>
```

```
          <speciesReference species="M_c3red_e" stoichiometry="1"/>
          <speciesReference species="M_h_e" stoichiometry="2"/>
        </listOfProducts>
        <kineticLaw>
          <math xmlns="http://www.w3.org/1998/Math/MathML">
            <ci> FLUX_VALUE </ci>
          </math>
          <listOfParameters>
            <parameter id="LOWER_BOUND" value="-100"
units="mmol_per_gDW_per_hr"/>
            <parameter id="UPPER_BOUND" value="100"
units="mmol_per_gDW_per_hr"/>
            <parameter id="FLUX_VALUE" value="0"
units="mmol_per_gDW_per_hr"/>
            <parameter id="OBJECTIVE_COEFFICIENT" value="0"
units="mmol_per_gDW_per_hr"/>
          </listOfParameters>
        </kineticLaw>
      </reaction>
      <reaction id="R_atp_synthase" name="2 h[e] + 1 adp[c] --> 2 h[c] + 1
atp[c]" reversible="1">
        <notes>
          <html:p>GENE_ASSOCIATION:  DVU0779</html:p>
          <html:p>SUBSYSTEM:  exchange_electron</html:p>
          <html:p>note: </html:p>
          <html:p>Equation: 2 h[e] + 1 adp[c] --> 2 h[c] + 1
atp[c]</html:p>
        </notes>
        <listOfReactants>
          <speciesReference species="M_h_e" stoichiometry="2"/>
          <speciesReference species="M_adp_c" stoichiometry="1"/>
        </listOfReactants>
        <listOfProducts>
          <speciesReference species="M_h_c" stoichiometry="2"/>
          <speciesReference species="M_atp_c" stoichiometry="1"/>
        </listOfProducts>
        <kineticLaw>
          <math xmlns="http://www.w3.org/1998/Math/MathML">
            <ci> FLUX_VALUE </ci>
          </math>
          <listOfParameters>
            <parameter id="LOWER_BOUND" value="-100"
units="mmol_per_gDW_per_hr"/>
            <parameter id="UPPER_BOUND" value="100"
units="mmol_per_gDW_per_hr"/>
            <parameter id="FLUX_VALUE" value="0"
units="mmol_per_gDW_per_hr"/>
            <parameter id="OBJECTIVE_COEFFICIENT" value="0"
units="mmol_per_gDW_per_hr"/>
          </listOfParameters>
        </kineticLaw>
      </reaction>
      <reaction id="R_phosphate_acetyltransferase" name="1 accoa[c] + 1
pi[c] = 1 actp[c] + 1 coa[c]" reversible="1">
        <notes>
          <html:p>GENE_ASSOCIATION:  DVU3029</html:p>
          <html:p>SUBSYSTEM:  fermentation_acetate</html:p>
          <html:p>note: </html:p>
          <html:p>Equation: 1 accoa[c] + 1 pi[c] = 1 actp[c] + 1
coa[c]</html:p>
        </notes>
```

```
        <listOfReactants>
          <speciesReference species="M_accoa_c" stoichiometry="1"/>
          <speciesReference species="M_pi_c" stoichiometry="1"/>
        </listOfReactants>
        <listOfProducts>
          <speciesReference species="M_actp_c" stoichiometry="1"/>
          <speciesReference species="M_coa_c" stoichiometry="1"/>
        </listOfProducts>
        <kineticLaw>
          <math xmlns="http://www.w3.org/1998/Math/MathML">
            <ci> FLUX_VALUE </ci>
          </math>
          <listOfParameters>
            <parameter id="LOWER_BOUND" value="0"
units="mmol_per_gDW_per_hr"/>
            <parameter id="UPPER_BOUND" value="100"
units="mmol_per_gDW_per_hr"/>
            <parameter id="FLUX_VALUE" value="0"
units="mmol_per_gDW_per_hr"/>
            <parameter id="OBJECTIVE_COEFFICIENT" value="0"
units="mmol_per_gDW_per_hr"/>
          </listOfParameters>
        </kineticLaw>
      </reaction>
      <reaction id="R_acetate_kinase" name="1 actp[c] + 1 adp[c] = 1 ac[c]
+ 1 atp[c]" reversible="1">
        <notes>
          <html:p>GENE_ASSOCIATION:  DVU3030</html:p>
          <html:p>SUBSYSTEM:  fermentation_acetate</html:p>
          <html:p>note: </html:p>
          <html:p>Equation: 1 actp[c] + 1 adp[c] = 1 ac[c] + 1
atp[c]</html:p>
        </notes>
        <listOfReactants>
          <speciesReference species="M_actp_c" stoichiometry="1"/>
          <speciesReference species="M_adp_c" stoichiometry="1"/>
        </listOfReactants>
        <listOfProducts>
          <speciesReference species="M_ac_c" stoichiometry="1"/>
          <speciesReference species="M_atp_c" stoichiometry="1"/>
        </listOfProducts>
        <kineticLaw>
          <math xmlns="http://www.w3.org/1998/Math/MathML">
            <ci> FLUX_VALUE </ci>
          </math>
          <listOfParameters>
            <parameter id="LOWER_BOUND" value="0"
units="mmol_per_gDW_per_hr"/>
            <parameter id="UPPER_BOUND" value="100"
units="mmol_per_gDW_per_hr"/>
            <parameter id="FLUX_VALUE" value="0"
units="mmol_per_gDW_per_hr"/>
            <parameter id="OBJECTIVE_COEFFICIENT" value="0"
units="mmol_per_gDW_per_hr"/>
          </listOfParameters>
        </kineticLaw>
      </reaction>
      <reaction id="R_acetyl_CoA_synthetase" name="1 ac[c] + 1 coa[c] + 1
atp[c] = 1 accoa[c] + 1 adp[c] + 1 ppi[c]" reversible="1">
        <notes>
          <html:p>GENE_ASSOCIATION:  DVU0748</html:p>
```

```
          <html:p>SUBSYSTEM:  fermentation_acetate</html:p>
          <html:p>note: </html:p>
          <html:p>Equation: 1 ac[c] + 1 coa[c] + 1 atp[c] = 1 accoa[c] + 1
adp[c] + 1 ppi[c]</html:p>
        </notes>
        <listOfReactants>
          <speciesReference species="M_ac_c" stoichiometry="1"/>
          <speciesReference species="M_coa_c" stoichiometry="1"/>
          <speciesReference species="M_atp_c" stoichiometry="1"/>
        </listOfReactants>
        <listOfProducts>
          <speciesReference species="M_accoa_c" stoichiometry="1"/>
          <speciesReference species="M_adp_c" stoichiometry="1"/>
          <speciesReference species="M_ppi_c" stoichiometry="1"/>
        </listOfProducts>
        <kineticLaw>
          <math xmlns="http://www.w3.org/1998/Math/MathML">
            <ci> FLUX_VALUE </ci>
          </math>
          <listOfParameters>
            <parameter id="LOWER_BOUND" value="0"
units="mmol_per_gDW_per_hr"/>
            <parameter id="UPPER_BOUND" value="100"
units="mmol_per_gDW_per_hr"/>
            <parameter id="FLUX_VALUE" value="0"
units="mmol_per_gDW_per_hr"/>
            <parameter id="OBJECTIVE_COEFFICIENT" value="0"
units="mmol_per_gDW_per_hr"/>
          </listOfParameters>
        </kineticLaw>
      </reaction>
      <reaction id="R_g6p_isomerase" name="1 g6p[c] = 1 f6p[c]"
reversible="1">
        <notes>
          <html:p>GENE_ASSOCIATION:  DVU3222</html:p>
          <html:p>SUBSYSTEM:  glycolysis</html:p>
          <html:p>note: </html:p>
          <html:p>Equation: 1 g6p[c] = 1 f6p[c]</html:p>
        </notes>
        <listOfReactants>
          <speciesReference species="M_g6p_c" stoichiometry="1"/>
        </listOfReactants>
        <listOfProducts>
          <speciesReference species="M_f6p_c" stoichiometry="1"/>
        </listOfProducts>
        <kineticLaw>
          <math xmlns="http://www.w3.org/1998/Math/MathML">
            <ci> FLUX_VALUE </ci>
          </math>
          <listOfParameters>
            <parameter id="LOWER_BOUND" value="-100"
units="mmol_per_gDW_per_hr"/>
            <parameter id="UPPER_BOUND" value="100"
units="mmol_per_gDW_per_hr"/>
            <parameter id="FLUX_VALUE" value="0"
units="mmol_per_gDW_per_hr"/>
            <parameter id="OBJECTIVE_COEFFICIENT" value="0"
units="mmol_per_gDW_per_hr"/>
          </listOfParameters>
        </kineticLaw>
      </reaction>
```

```
      <reaction id="R_6phosphofructokinase" name="1 atp[c] + 1 f6p[c] --> 1
adp[c] + 1 fbp[c]" reversible="0">
        <notes>
          <html:p>GENE_ASSOCIATION:  DVU2061</html:p>
          <html:p>SUBSYSTEM:  glycolysis</html:p>
          <html:p>note: </html:p>
          <html:p>Equation: 1 atp[c] + 1 f6p[c] --> 1 adp[c] + 1
fbp[c]</html:p>
        </notes>
        <listOfReactants>
          <speciesReference species="M_atp_c" stoichiometry="1"/>
          <speciesReference species="M_f6p_c" stoichiometry="1"/>
        </listOfReactants>
        <listOfProducts>
          <speciesReference species="M_adp_c" stoichiometry="1"/>
          <speciesReference species="M_fbp_c" stoichiometry="1"/>
        </listOfProducts>
        <kineticLaw>
          <math xmlns="http://www.w3.org/1998/Math/MathML">
            <ci> FLUX_VALUE </ci>
          </math>
          <listOfParameters>
            <parameter id="LOWER_BOUND" value="0"
units="mmol_per_gDW_per_hr"/>
            <parameter id="UPPER_BOUND" value="0"
units="mmol_per_gDW_per_hr"/>
            <parameter id="FLUX_VALUE" value="0"
units="mmol_per_gDW_per_hr"/>
            <parameter id="OBJECTIVE_COEFFICIENT" value="0"
units="mmol_per_gDW_per_hr"/>
          </listOfParameters>
        </kineticLaw>
      </reaction>
      <reaction id="R_fructose_bisphosphatase" name="1 fbp[c] + 1 h2o[c] --
> 1 f6p[c] + 1 pi[c]" reversible="0">
        <notes>
          <html:p>GENE_ASSOCIATION:  DVU1539</html:p>
          <html:p>SUBSYSTEM:  glycolysis</html:p>
          <html:p>note: </html:p>
          <html:p>Equation: 1 fbp[c] + 1 h2o[c] --> 1 f6p[c] + 1
pi[c]</html:p>
        </notes>
        <listOfReactants>
          <speciesReference species="M_fbp_c" stoichiometry="1"/>
          <speciesReference species="M_h2o_c" stoichiometry="1"/>
        </listOfReactants>
        <listOfProducts>
          <speciesReference species="M_f6p_c" stoichiometry="1"/>
          <speciesReference species="M_pi_c" stoichiometry="1"/>
        </listOfProducts>
        <kineticLaw>
          <math xmlns="http://www.w3.org/1998/Math/MathML">
            <ci> FLUX_VALUE </ci>
          </math>
          <listOfParameters>
            <parameter id="LOWER_BOUND" value="-100"
units="mmol_per_gDW_per_hr"/>
            <parameter id="UPPER_BOUND" value="100"
units="mmol_per_gDW_per_hr"/>
            <parameter id="FLUX_VALUE" value="0"
units="mmol_per_gDW_per_hr"/>
```

```
            <parameter id="OBJECTIVE_COEFFICIENT" value="0"
units="mmol_per_gDW_per_hr"/>
          </listOfParameters>
        </kineticLaw>
      </reaction>
      <reaction id="R_fructose_bisphosphate_aldolase" name="1 fbp[c] = 1
dhap[c] + 1 g3p[c]" reversible="1">
        <notes>
          <html:p>GENE_ASSOCIATION:  DVU0460</html:p>
          <html:p>SUBSYSTEM:  glycolysis</html:p>
          <html:p>note: </html:p>
          <html:p>Equation: 1 fbp[c] = 1 dhap[c] + 1 g3p[c]</html:p>
        </notes>
        <listOfReactants>
          <speciesReference species="M_fbp_c" stoichiometry="1"/>
        </listOfReactants>
        <listOfProducts>
          <speciesReference species="M_dhap_c" stoichiometry="1"/>
          <speciesReference species="M_g3p_c" stoichiometry="1"/>
        </listOfProducts>
        <kineticLaw>
          <math xmlns="http://www.w3.org/1998/Math/MathML">
            <ci> FLUX_VALUE </ci>
          </math>
          <listOfParameters>
            <parameter id="LOWER_BOUND" value="-100"
units="mmol_per_gDW_per_hr"/>
            <parameter id="UPPER_BOUND" value="100"
units="mmol_per_gDW_per_hr"/>
            <parameter id="FLUX_VALUE" value="0"
units="mmol_per_gDW_per_hr"/>
            <parameter id="OBJECTIVE_COEFFICIENT" value="0"
units="mmol_per_gDW_per_hr"/>
          </listOfParameters>
        </kineticLaw>
      </reaction>
      <reaction id="R_triose_phosphate_isomerase" name="1 dhap[c] = 1
g3p[c]" reversible="1">
        <notes>
          <html:p>GENE_ASSOCIATION:  DVU1677</html:p>
          <html:p>SUBSYSTEM:  glycolysis</html:p>
          <html:p>note: </html:p>
          <html:p>Equation: 1 dhap[c] = 1 g3p[c]</html:p>
        </notes>
        <listOfReactants>
          <speciesReference species="M_dhap_c" stoichiometry="1"/>
        </listOfReactants>
        <listOfProducts>
          <speciesReference species="M_g3p_c" stoichiometry="1"/>
        </listOfProducts>
        <kineticLaw>
          <math xmlns="http://www.w3.org/1998/Math/MathML">
            <ci> FLUX_VALUE </ci>
          </math>
          <listOfParameters>
            <parameter id="LOWER_BOUND" value="-100"
units="mmol_per_gDW_per_hr"/>
            <parameter id="UPPER_BOUND" value="100"
units="mmol_per_gDW_per_hr"/>
            <parameter id="FLUX_VALUE" value="0"
units="mmol_per_gDW_per_hr"/>
```

```
            <parameter id="OBJECTIVE_COEFFICIENT" value="0"
units="mmol_per_gDW_per_hr"/>
          </listOfParameters>
        </kineticLaw>
      </reaction>
      <reaction id="R_glyceraldehyde_3_phosphate_dehydrogenase" name="1
g3p[c] + 1 nad[c] + 1 pi[c] = 1 13bpg[c] + 1 nadh[c]" reversible="1">
        <notes>
          <html:p>GENE_ASSOCIATION:  DVU0565</html:p>
          <html:p>SUBSYSTEM:  glycolysis</html:p>
          <html:p>note: </html:p>
          <html:p>Equation: 1 g3p[c] + 1 nad[c] + 1 pi[c] = 1 13bpg[c] + 1
nadh[c]</html:p>
        </notes>
        <listOfReactants>
          <speciesReference species="M_g3p_c" stoichiometry="1"/>
          <speciesReference species="M_nad_c" stoichiometry="1"/>
          <speciesReference species="M_pi_c" stoichiometry="1"/>
        </listOfReactants>
        <listOfProducts>
          <speciesReference species="M_13bpg_c" stoichiometry="1"/>
          <speciesReference species="M_nadh_c" stoichiometry="1"/>
        </listOfProducts>
        <kineticLaw>
          <math xmlns="http://www.w3.org/1998/Math/MathML">
            <ci> FLUX_VALUE </ci>
          </math>
          <listOfParameters>
            <parameter id="LOWER_BOUND" value="-100"
units="mmol_per_gDW_per_hr"/>
            <parameter id="UPPER_BOUND" value="100"
units="mmol_per_gDW_per_hr"/>
            <parameter id="FLUX_VALUE" value="0"
units="mmol_per_gDW_per_hr"/>
            <parameter id="OBJECTIVE_COEFFICIENT" value="0"
units="mmol_per_gDW_per_hr"/>
          </listOfParameters>
        </kineticLaw>
      </reaction>
      <reaction id="R_phosphoglycerate_kinase" name="1 13bpg[c] + 1 adp[c]
= 1 3pg[c] + 1 atp[c]" reversible="1">
        <notes>
          <html:p>GENE_ASSOCIATION:  DVU2529</html:p>
          <html:p>SUBSYSTEM:  glycolysis</html:p>
          <html:p>note: </html:p>
          <html:p>Equation: 1 13bpg[c] + 1 adp[c] = 1 3pg[c] + 1
atp[c]</html:p>
        </notes>
        <listOfReactants>
          <speciesReference species="M_13bpg_c" stoichiometry="1"/>
          <speciesReference species="M_adp_c" stoichiometry="1"/>
        </listOfReactants>
        <listOfProducts>
          <speciesReference species="M_3pg_c" stoichiometry="1"/>
          <speciesReference species="M_atp_c" stoichiometry="1"/>
        </listOfProducts>
        <kineticLaw>
          <math xmlns="http://www.w3.org/1998/Math/MathML">
            <ci> FLUX_VALUE </ci>
          </math>
          <listOfParameters>
```

```
            <parameter id="LOWER_BOUND" value="-100"
units="mmol_per_gDW_per_hr"/>
            <parameter id="UPPER_BOUND" value="100"
units="mmol_per_gDW_per_hr"/>
            <parameter id="FLUX_VALUE" value="0"
units="mmol_per_gDW_per_hr"/>
            <parameter id="OBJECTIVE_COEFFICIENT" value="0"
units="mmol_per_gDW_per_hr"/>
          </listOfParameters>
        </kineticLaw>
      </reaction>
      <reaction id="R_phosphoglycerate_mutase" name="1 3pg[c] = 1 2pg[c]"
reversible="1">
        <notes>
          <html:p>GENE_ASSOCIATION:  DVU0889</html:p>
          <html:p>SUBSYSTEM:  glycolysis</html:p>
          <html:p>note: </html:p>
          <html:p>Equation: 1 3pg[c] = 1 2pg[c]</html:p>
        </notes>
        <listOfReactants>
          <speciesReference species="M_3pg_c" stoichiometry="1"/>
        </listOfReactants>
        <listOfProducts>
          <speciesReference species="M_2pg_c" stoichiometry="1"/>
        </listOfProducts>
        <kineticLaw>
          <math xmlns="http://www.w3.org/1998/Math/MathML">
            <ci> FLUX_VALUE </ci>
          </math>
          <listOfParameters>
            <parameter id="LOWER_BOUND" value="-100"
units="mmol_per_gDW_per_hr"/>
            <parameter id="UPPER_BOUND" value="100"
units="mmol_per_gDW_per_hr"/>
            <parameter id="FLUX_VALUE" value="0"
units="mmol_per_gDW_per_hr"/>
            <parameter id="OBJECTIVE_COEFFICIENT" value="0"
units="mmol_per_gDW_per_hr"/>
          </listOfParameters>
        </kineticLaw>
      </reaction>
      <reaction id="R_phosphopyruvate_hydratase" name="1 2pg[c] = 1 pep[c]
+ 1 h2o[c]" reversible="1">
        <notes>
          <html:p>GENE_ASSOCIATION:  DVU0322</html:p>
          <html:p>SUBSYSTEM:  glycolysis</html:p>
          <html:p>note: </html:p>
          <html:p>Equation: 1 2pg[c] = 1 pep[c] + 1 h2o[c]</html:p>
        </notes>
        <listOfReactants>
          <speciesReference species="M_2pg_c" stoichiometry="1"/>
        </listOfReactants>
        <listOfProducts>
          <speciesReference species="M_pep_c" stoichiometry="1"/>
          <speciesReference species="M_h2o_c" stoichiometry="1"/>
        </listOfProducts>
        <kineticLaw>
          <math xmlns="http://www.w3.org/1998/Math/MathML">
            <ci> FLUX_VALUE </ci>
          </math>
          <listOfParameters>
```

```
            <parameter id="LOWER_BOUND" value="-100"
units="mmol_per_gDW_per_hr"/>
            <parameter id="UPPER_BOUND" value="100"
units="mmol_per_gDW_per_hr"/>
            <parameter id="FLUX_VALUE" value="0"
units="mmol_per_gDW_per_hr"/>
            <parameter id="OBJECTIVE_COEFFICIENT" value="0"
units="mmol_per_gDW_per_hr"/>
          </listOfParameters>
        </kineticLaw>
      </reaction>
      <reaction id="R_pyruvate_water_dikinase" name="1 adp[c] + 1 pi[c] + 1
pep[c] = 1 atp[c] + 1 h2o[c] + 1 pyr[c]" reversible="1">
        <notes>
          <html:p>GENE_ASSOCIATION:  DVU2514</html:p>
          <html:p>SUBSYSTEM:  glycolysis</html:p>
          <html:p>note: </html:p>
          <html:p>Equation: 1 adp[c] + 1 pi[c] + 1 pep[c] = 1 atp[c] + 1
h2o[c] + 1 pyr[c]</html:p>
        </notes>
        <listOfReactants>
          <speciesReference species="M_adp_c" stoichiometry="1"/>
          <speciesReference species="M_pi_c" stoichiometry="1"/>
          <speciesReference species="M_pep_c" stoichiometry="1"/>
        </listOfReactants>
        <listOfProducts>
          <speciesReference species="M_atp_c" stoichiometry="1"/>
          <speciesReference species="M_h2o_c" stoichiometry="1"/>
          <speciesReference species="M_pyr_c" stoichiometry="1"/>
        </listOfProducts>
        <kineticLaw>
          <math xmlns="http://www.w3.org/1998/Math/MathML">
            <ci> FLUX_VALUE </ci>
          </math>
          <listOfParameters>
            <parameter id="LOWER_BOUND" value="-100"
units="mmol_per_gDW_per_hr"/>
            <parameter id="UPPER_BOUND" value="100"
units="mmol_per_gDW_per_hr"/>
            <parameter id="FLUX_VALUE" value="0"
units="mmol_per_gDW_per_hr"/>
            <parameter id="OBJECTIVE_COEFFICIENT" value="0"
units="mmol_per_gDW_per_hr"/>
          </listOfParameters>
        </kineticLaw>
      </reaction>
      <reaction id="R_L_lactate_dehydrogenase" name="1 lac[c] + 1 nad[c] --
> 1 pyr[c] + 1 nadh[c]" reversible="1">
        <notes>
          <html:p>GENE_ASSOCIATION:  DVU0600</html:p>
          <html:p>SUBSYSTEM:  lactate_in_acetyl-coa</html:p>
          <html:p>note: </html:p>
          <html:p>Equation: 1 lac[c] + 1 nad[c] --> 1 pyr[c] + 1
nadh[c]</html:p>
        </notes>
        <listOfReactants>
          <speciesReference species="M_lac_c" stoichiometry="1"/>
          <speciesReference species="M_nad_c" stoichiometry="1"/>
        </listOfReactants>
        <listOfProducts>
          <speciesReference species="M_pyr_c" stoichiometry="1"/>
```

```
          <speciesReference species="M_nadh_c" stoichiometry="1"/>
        </listOfProducts>
        <kineticLaw>
          <math xmlns="http://www.w3.org/1998/Math/MathML">
            <ci> FLUX_VALUE </ci>
          </math>
          <listOfParameters>
            <parameter id="LOWER_BOUND" value="-100"
units="mmol_per_gDW_per_hr"/>
            <parameter id="UPPER_BOUND" value="100"
units="mmol_per_gDW_per_hr"/>
            <parameter id="FLUX_VALUE" value="0"
units="mmol_per_gDW_per_hr"/>
            <parameter id="OBJECTIVE_COEFFICIENT" value="0"
units="mmol_per_gDW_per_hr"/>
          </listOfParameters>
        </kineticLaw>
      </reaction>
      <reaction id="R_pyruvate_ferredoxin_oxidoreductase" name="1 pyr[c] +
1 coa[c] + 1 fedox[c] = 1 accoa[c] + 1 co2[c] + 1 fedred[c]"
reversible="1">
        <notes>
          <html:p>GENE_ASSOCIATION:  DVU3025</html:p>
          <html:p>SUBSYSTEM:  lactate_in_acetyl-coa</html:p>
          <html:p>note: </html:p>
          <html:p>Equation: 1 pyr[c] + 1 coa[c] + 1 fedox[c] = 1 accoa[c] +
1 co2[c] + 1 fedred[c]</html:p>
        </notes>
        <listOfReactants>
          <speciesReference species="M_pyr_c" stoichiometry="1"/>
          <speciesReference species="M_coa_c" stoichiometry="1"/>
          <speciesReference species="M_fedox_c" stoichiometry="1"/>
        </listOfReactants>
        <listOfProducts>
          <speciesReference species="M_accoa_c" stoichiometry="1"/>
          <speciesReference species="M_co2_c" stoichiometry="1"/>
          <speciesReference species="M_fedred_c" stoichiometry="1"/>
        </listOfProducts>
        <kineticLaw>
          <math xmlns="http://www.w3.org/1998/Math/MathML">
            <ci> FLUX_VALUE </ci>
          </math>
          <listOfParameters>
            <parameter id="LOWER_BOUND" value="-100"
units="mmol_per_gDW_per_hr"/>
            <parameter id="UPPER_BOUND" value="100"
units="mmol_per_gDW_per_hr"/>
            <parameter id="FLUX_VALUE" value="0"
units="mmol_per_gDW_per_hr"/>
            <parameter id="OBJECTIVE_COEFFICIENT" value="0"
units="mmol_per_gDW_per_hr"/>
          </listOfParameters>
        </kineticLaw>
      </reaction>
      <reaction id="R_transketolase" name="1 s7p[c] + 1 g3p[c] = 1
ribo5p[c] + 1 xyl5p[c]" reversible="1">
        <notes>
          <html:p>GENE_ASSOCIATION:  DVU2530</html:p>
          <html:p>SUBSYSTEM:  pentose_phosphate</html:p>
          <html:p>note: </html:p>
```

```
          <html:p>Equation: 1 s7p[c] + 1 g3p[c] = 1 ribo5p[c] + 1
xyl5p[c]</html:p>
        </notes>
        <listOfReactants>
          <speciesReference species="M_s7p_c" stoichiometry="1"/>
          <speciesReference species="M_g3p_c" stoichiometry="1"/>
        </listOfReactants>
        <listOfProducts>
          <speciesReference species="M_ribo5p_c" stoichiometry="1"/>
          <speciesReference species="M_xyl5p_c" stoichiometry="1"/>
        </listOfProducts>
        <kineticLaw>
          <math xmlns="http://www.w3.org/1998/Math/MathML">
            <ci> FLUX_VALUE </ci>
          </math>
          <listOfParameters>
            <parameter id="LOWER_BOUND" value="-100"
units="mmol_per_gDW_per_hr"/>
            <parameter id="UPPER_BOUND" value="100"
units="mmol_per_gDW_per_hr"/>
            <parameter id="FLUX_VALUE" value="0"
units="mmol_per_gDW_per_hr"/>
            <parameter id="OBJECTIVE_COEFFICIENT" value="0"
units="mmol_per_gDW_per_hr"/>
          </listOfParameters>
        </kineticLaw>
      </reaction>
      <reaction id="R_transaldolase" name="1 s7p[c] + 1 g3p[c] = 1 e4p[c] +
1 f6p[c]" reversible="1">
        <notes>
          <html:p>GENE_ASSOCIATION:  DVU1658</html:p>
          <html:p>SUBSYSTEM:  pentose_phosphate</html:p>
          <html:p>note: </html:p>
          <html:p>Equation: 1 s7p[c] + 1 g3p[c] = 1 e4p[c] + 1
f6p[c]</html:p>
        </notes>
        <listOfReactants>
          <speciesReference species="M_s7p_c" stoichiometry="1"/>
          <speciesReference species="M_g3p_c" stoichiometry="1"/>
        </listOfReactants>
        <listOfProducts>
          <speciesReference species="M_e4p_c" stoichiometry="1"/>
          <speciesReference species="M_f6p_c" stoichiometry="1"/>
        </listOfProducts>
        <kineticLaw>
          <math xmlns="http://www.w3.org/1998/Math/MathML">
            <ci> FLUX_VALUE </ci>
          </math>
          <listOfParameters>
            <parameter id="LOWER_BOUND" value="-100"
units="mmol_per_gDW_per_hr"/>
            <parameter id="UPPER_BOUND" value="100"
units="mmol_per_gDW_per_hr"/>
            <parameter id="FLUX_VALUE" value="0"
units="mmol_per_gDW_per_hr"/>
            <parameter id="OBJECTIVE_COEFFICIENT" value="0"
units="mmol_per_gDW_per_hr"/>
          </listOfParameters>
        </kineticLaw>
      </reaction>
```

```
      <reaction id="R_transketolase_tkt" name="1 e4p[c] + 1 xyl5p[c] = 1
f6p[c] + 1 g3p[c]" reversible="1">
        <notes>
          <html:p>GENE_ASSOCIATION:  DVU2530</html:p>
          <html:p>SUBSYSTEM:  pentose_phosphate</html:p>
          <html:p>note: </html:p>
          <html:p>Equation: 1 e4p[c] + 1 xyl5p[c] = 1 f6p[c] + 1
g3p[c]</html:p>
        </notes>
        <listOfReactants>
          <speciesReference species="M_e4p_c" stoichiometry="1"/>
          <speciesReference species="M_xyl5p_c" stoichiometry="1"/>
        </listOfReactants>
        <listOfProducts>
          <speciesReference species="M_f6p_c" stoichiometry="1"/>
          <speciesReference species="M_g3p_c" stoichiometry="1"/>
        </listOfProducts>
        <kineticLaw>
          <math xmlns="http://www.w3.org/1998/Math/MathML">
            <ci> FLUX_VALUE </ci>
          </math>
          <listOfParameters>
            <parameter id="LOWER_BOUND" value="-100"
units="mmol_per_gDW_per_hr"/>
            <parameter id="UPPER_BOUND" value="100"
units="mmol_per_gDW_per_hr"/>
            <parameter id="FLUX_VALUE" value="0"
units="mmol_per_gDW_per_hr"/>
            <parameter id="OBJECTIVE_COEFFICIENT" value="0"
units="mmol_per_gDW_per_hr"/>
          </listOfParameters>
        </kineticLaw>
      </reaction>
      <reaction id="R_ribulose_phosphate_3_epimerase" name="1 ribu5p[c] = 1
xyl5p[c]" reversible="1">
        <notes>
          <html:p>GENE_ASSOCIATION:  DVU2531</html:p>
          <html:p>SUBSYSTEM:  pentose_phosphate</html:p>
          <html:p>note: </html:p>
          <html:p>Equation: 1 ribu5p[c] = 1 xyl5p[c]</html:p>
        </notes>
        <listOfReactants>
          <speciesReference species="M_ribu5p_c" stoichiometry="1"/>
        </listOfReactants>
        <listOfProducts>
          <speciesReference species="M_xyl5p_c" stoichiometry="1"/>
        </listOfProducts>
        <kineticLaw>
          <math xmlns="http://www.w3.org/1998/Math/MathML">
            <ci> FLUX_VALUE </ci>
          </math>
          <listOfParameters>
            <parameter id="LOWER_BOUND" value="-100"
units="mmol_per_gDW_per_hr"/>
            <parameter id="UPPER_BOUND" value="100"
units="mmol_per_gDW_per_hr"/>
            <parameter id="FLUX_VALUE" value="0"
units="mmol_per_gDW_per_hr"/>
            <parameter id="OBJECTIVE_COEFFICIENT" value="0"
units="mmol_per_gDW_per_hr"/>
          </listOfParameters>
```

```
        </kineticLaw>
      </reaction>
      <reaction id="R_ribose_5_phosphate_isomerase" name="1 ribu5p[c] = 1
ribo5p[c]" reversible="1">
        <notes>
          <html:p>GENE_ASSOCIATION:  DVU1580</html:p>
          <html:p>SUBSYSTEM:  pentose_phosphate</html:p>
          <html:p>note: </html:p>
          <html:p>Equation: 1 ribu5p[c] = 1 ribo5p[c]</html:p>
        </notes>
        <listOfReactants>
          <speciesReference species="M_ribu5p_c" stoichiometry="1"/>
        </listOfReactants>
        <listOfProducts>
          <speciesReference species="M_ribo5p_c" stoichiometry="1"/>
        </listOfProducts>
        <kineticLaw>
          <math xmlns="http://www.w3.org/1998/Math/MathML">
            <ci> FLUX_VALUE </ci>
          </math>
          <listOfParameters>
            <parameter id="LOWER_BOUND" value="-100"
units="mmol_per_gDW_per_hr"/>
            <parameter id="UPPER_BOUND" value="100"
units="mmol_per_gDW_per_hr"/>
            <parameter id="FLUX_VALUE" value="0"
units="mmol_per_gDW_per_hr"/>
            <parameter id="OBJECTIVE_COEFFICIENT" value="0"
units="mmol_per_gDW_per_hr"/>
          </listOfParameters>
        </kineticLaw>
      </reaction>
      <reaction id="R_AMP_phosphotransferase" name="1 amp[c] + 1 atp[c] = 2
adp[c]" reversible="1">
        <notes>
          <html:p>GENE_ASSOCIATION:  DVU1932</html:p>
          <html:p>SUBSYSTEM:  recycle_amp</html:p>
          <html:p>note: </html:p>
          <html:p>Equation: 1 amp[c] + 1 atp[c] = 2 adp[c]</html:p>
        </notes>
        <listOfReactants>
          <speciesReference species="M_amp_c" stoichiometry="1"/>
          <speciesReference species="M_atp_c" stoichiometry="1"/>
        </listOfReactants>
        <listOfProducts>
          <speciesReference species="M_adp_c" stoichiometry="2"/>
        </listOfProducts>
        <kineticLaw>
          <math xmlns="http://www.w3.org/1998/Math/MathML">
            <ci> FLUX_VALUE </ci>
          </math>
          <listOfParameters>
            <parameter id="LOWER_BOUND" value="-100"
units="mmol_per_gDW_per_hr"/>
            <parameter id="UPPER_BOUND" value="100"
units="mmol_per_gDW_per_hr"/>
            <parameter id="FLUX_VALUE" value="0"
units="mmol_per_gDW_per_hr"/>
            <parameter id="OBJECTIVE_COEFFICIENT" value="0"
units="mmol_per_gDW_per_hr"/>
          </listOfParameters>
```

```
        </kineticLaw>
      </reaction>
      <reaction id="R_citrate_Re_synthase" name="1 accoa[c] + 1 h2o[c] + 1
oaa[c] = 1 cit[c] + 1 coa[c]" reversible="1">
        <notes>
          <html:p>GENE_ASSOCIATION:  DVU0398</html:p>
          <html:p>SUBSYSTEM:  TCA</html:p>
          <html:p>note: </html:p>
          <html:p>Equation: 1 accoa[c] + 1 h2o[c] + 1 oaa[c] = 1 cit[c] + 1
coa[c]</html:p>
        </notes>
        <listOfReactants>
          <speciesReference species="M_accoa_c" stoichiometry="1"/>
          <speciesReference species="M_h2o_c" stoichiometry="1"/>
          <speciesReference species="M_oaa_c" stoichiometry="1"/>
        </listOfReactants>
        <listOfProducts>
          <speciesReference species="M_cit_c" stoichiometry="1"/>
          <speciesReference species="M_coa_c" stoichiometry="1"/>
        </listOfProducts>
        <kineticLaw>
          <math xmlns="http://www.w3.org/1998/Math/MathML">
            <ci> FLUX_VALUE </ci>
          </math>
          <listOfParameters>
            <parameter id="LOWER_BOUND" value="-100"
units="mmol_per_gDW_per_hr"/>
            <parameter id="UPPER_BOUND" value="100"
units="mmol_per_gDW_per_hr"/>
            <parameter id="FLUX_VALUE" value="0"
units="mmol_per_gDW_per_hr"/>
            <parameter id="OBJECTIVE_COEFFICIENT" value="0"
units="mmol_per_gDW_per_hr"/>
          </listOfParameters>
        </kineticLaw>
      </reaction>
      <reaction id="R_aconitate_hydratase" name="1 cit[c] = 1 icit[c]"
reversible="1">
        <notes>
          <html:p>GENE_ASSOCIATION:  DVU1064</html:p>
          <html:p>SUBSYSTEM:  TCA</html:p>
          <html:p>note: </html:p>
          <html:p>Equation: 1 cit[c] = 1 icit[c]</html:p>
        </notes>
        <listOfReactants>
          <speciesReference species="M_cit_c" stoichiometry="1"/>
        </listOfReactants>
        <listOfProducts>
          <speciesReference species="M_icit_c" stoichiometry="1"/>
        </listOfProducts>
        <kineticLaw>
          <math xmlns="http://www.w3.org/1998/Math/MathML">
            <ci> FLUX_VALUE </ci>
          </math>
          <listOfParameters>
            <parameter id="LOWER_BOUND" value="-100"
units="mmol_per_gDW_per_hr"/>
            <parameter id="UPPER_BOUND" value="100"
units="mmol_per_gDW_per_hr"/>
            <parameter id="FLUX_VALUE" value="0"
units="mmol_per_gDW_per_hr"/>
```

```
            <parameter id="OBJECTIVE_COEFFICIENT" value="0"
units="mmol_per_gDW_per_hr"/>
          </listOfParameters>
        </kineticLaw>
      </reaction>
      <reaction id="R_isocitrate_dehydrogenase" name="1 icit[c] + 1 nad[c]
= 1 oxo[c] + 1 nadh[c] + 1 co2[c]" reversible="1">
        <notes>
          <html:p>GENE_ASSOCIATION:  DVU0477</html:p>
          <html:p>SUBSYSTEM:  TCA</html:p>
          <html:p>note: </html:p>
          <html:p>Equation: 1 icit[c] + 1 nad[c] = 1 oxo[c] + 1 nadh[c] + 1
co2[c]</html:p>
        </notes>
        <listOfReactants>
          <speciesReference species="M_icit_c" stoichiometry="1"/>
          <speciesReference species="M_nad_c" stoichiometry="1"/>
        </listOfReactants>
        <listOfProducts>
          <speciesReference species="M_oxo_c" stoichiometry="1"/>
          <speciesReference species="M_nadh_c" stoichiometry="1"/>
          <speciesReference species="M_co2_c" stoichiometry="1"/>
        </listOfProducts>
        <kineticLaw>
          <math xmlns="http://www.w3.org/1998/Math/MathML">
            <ci> FLUX_VALUE </ci>
          </math>
          <listOfParameters>
            <parameter id="LOWER_BOUND" value="-100"
units="mmol_per_gDW_per_hr"/>
            <parameter id="UPPER_BOUND" value="100"
units="mmol_per_gDW_per_hr"/>
            <parameter id="FLUX_VALUE" value="0"
units="mmol_per_gDW_per_hr"/>
            <parameter id="OBJECTIVE_COEFFICIENT" value="0"
units="mmol_per_gDW_per_hr"/>
          </listOfParameters>
        </kineticLaw>
      </reaction>
      <reaction id="R_2_ketoglutarate_ferredoxin_oxidoreductase" name="1
oxo[c] + 1 coa[c] + 1 fedox[c] = 1 succoa[c] + 1 co2[c] + 1 fedred[c] + 2
h[c]" reversible="1">
        <notes>
          <html:p>GENE_ASSOCIATION:  DVU1569</html:p>
          <html:p>SUBSYSTEM:  TCA</html:p>
          <html:p>note: </html:p>
          <html:p>Equation: 1 oxo[c] + 1 coa[c] + 1 fedox[c] = 1 succoa[c]
+ 1 co2[c] + 1 fedred[c] + 2 h[c]</html:p>
        </notes>
        <listOfReactants>
          <speciesReference species="M_oxo_c" stoichiometry="1"/>
          <speciesReference species="M_coa_c" stoichiometry="1"/>
          <speciesReference species="M_fedox_c" stoichiometry="1"/>
        </listOfReactants>
        <listOfProducts>
          <speciesReference species="M_succoa_c" stoichiometry="1"/>
          <speciesReference species="M_co2_c" stoichiometry="1"/>
          <speciesReference species="M_fedred_c" stoichiometry="1"/>
          <speciesReference species="M_h_c" stoichiometry="2"/>
        </listOfProducts>
        <kineticLaw>
```

```
          <math xmlns="http://www.w3.org/1998/Math/MathML">
            <ci> FLUX_VALUE </ci>
          </math>
          <listOfParameters>
            <parameter id="LOWER_BOUND" value="0"
units="mmol_per_gDW_per_hr"/>
            <parameter id="UPPER_BOUND" value="0"
units="mmol_per_gDW_per_hr"/>
            <parameter id="FLUX_VALUE" value="0"
units="mmol_per_gDW_per_hr"/>
            <parameter id="OBJECTIVE_COEFFICIENT" value="0"
units="mmol_per_gDW_per_hr"/>
          </listOfParameters>
        </kineticLaw>
      </reaction>
      <reaction id="R_succinate_CoA_ligase" name="1 succoa[c] + 1 adp[c] =
1 succ[c] + 1 atp[c] + 1 coa[c]" reversible="1">
        <notes>
          <html:p>GENE_ASSOCIATION:  DVU2137</html:p>
          <html:p>SUBSYSTEM:  TCA</html:p>
          <html:p>note: </html:p>
          <html:p>Equation: 1 succoa[c] + 1 adp[c] = 1 succ[c] + 1 atp[c] +
1 coa[c]</html:p>
        </notes>
        <listOfReactants>
          <speciesReference species="M_succoa_c" stoichiometry="1"/>
          <speciesReference species="M_adp_c" stoichiometry="1"/>
        </listOfReactants>
        <listOfProducts>
          <speciesReference species="M_succ_c" stoichiometry="1"/>
          <speciesReference species="M_atp_c" stoichiometry="1"/>
          <speciesReference species="M_coa_c" stoichiometry="1"/>
        </listOfProducts>
        <kineticLaw>
          <math xmlns="http://www.w3.org/1998/Math/MathML">
            <ci> FLUX_VALUE </ci>
          </math>
          <listOfParameters>
            <parameter id="LOWER_BOUND" value="0"
units="mmol_per_gDW_per_hr"/>
            <parameter id="UPPER_BOUND" value="0"
units="mmol_per_gDW_per_hr"/>
            <parameter id="FLUX_VALUE" value="0"
units="mmol_per_gDW_per_hr"/>
            <parameter id="OBJECTIVE_COEFFICIENT" value="0"
units="mmol_per_gDW_per_hr"/>
          </listOfParameters>
        </kineticLaw>
      </reaction>
      <reaction id="R_quinone_deshydrogenase" name="1 quinone[c] = 1
quinol[c]" reversible="1">
        <notes>
          <html:p>GENE_ASSOCIATION:  </html:p>
          <html:p>SUBSYSTEM:  TCA</html:p>
          <html:p>note: </html:p>
          <html:p>Equation: 1 quinone[c] = 1 quinol[c]</html:p>
        </notes>
        <listOfReactants>
          <speciesReference species="M_quinone_c" stoichiometry="1"/>
        </listOfReactants>
        <listOfProducts>
```

```
          <speciesReference species="M_quinol_c" stoichiometry="1"/>
        </listOfProducts>
        <kineticLaw>
          <math xmlns="http://www.w3.org/1998/Math/MathML">
            <ci> FLUX_VALUE </ci>
          </math>
          <listOfParameters>
            <parameter id="LOWER_BOUND" value="-100"
units="mmol_per_gDW_per_hr"/>
            <parameter id="UPPER_BOUND" value="100"
units="mmol_per_gDW_per_hr"/>
            <parameter id="FLUX_VALUE" value="0"
units="mmol_per_gDW_per_hr"/>
            <parameter id="OBJECTIVE_COEFFICIENT" value="0"
units="mmol_per_gDW_per_hr"/>
          </listOfParameters>
        </kineticLaw>
      </reaction>
      <reaction id="R_succinate_dehydrogenase" name="1 succ[c] + 1
quinone[c] = 1 fum[c] + 1 quinol[c]" reversible="1">
        <notes>
          <html:p>GENE_ASSOCIATION:  DVU2674</html:p>
          <html:p>SUBSYSTEM:  TCA</html:p>
          <html:p>note: </html:p>
          <html:p>Equation: 1 succ[c] + 1 quinone[c] = 1 fum[c] + 1
quinol[c]</html:p>
        </notes>
        <listOfReactants>
          <speciesReference species="M_succ_c" stoichiometry="1"/>
          <speciesReference species="M_quinone_c" stoichiometry="1"/>
        </listOfReactants>
        <listOfProducts>
          <speciesReference species="M_fum_c" stoichiometry="1"/>
          <speciesReference species="M_quinol_c" stoichiometry="1"/>
        </listOfProducts>
        <kineticLaw>
          <math xmlns="http://www.w3.org/1998/Math/MathML">
            <ci> FLUX_VALUE </ci>
          </math>
          <listOfParameters>
            <parameter id="LOWER_BOUND" value="-100"
units="mmol_per_gDW_per_hr"/>
            <parameter id="UPPER_BOUND" value="100"
units="mmol_per_gDW_per_hr"/>
            <parameter id="FLUX_VALUE" value="0"
units="mmol_per_gDW_per_hr"/>
            <parameter id="OBJECTIVE_COEFFICIENT" value="0"
units="mmol_per_gDW_per_hr"/>
          </listOfParameters>
        </kineticLaw>
      </reaction>
      <reaction id="R_fumarate_hydratase" name="1 mal[c] = 1 fum[c] + 1
h2o[c]" reversible="1">
        <notes>
          <html:p>GENE_ASSOCIATION:  DVU0080</html:p>
          <html:p>SUBSYSTEM:  TCA</html:p>
          <html:p>note: </html:p>
          <html:p>Equation: 1 mal[c] = 1 fum[c] + 1 h2o[c]</html:p>
        </notes>
        <listOfReactants>
          <speciesReference species="M_mal_c" stoichiometry="1"/>
```

```
        </listOfReactants>
        <listOfProducts>
          <speciesReference species="M_fum_c" stoichiometry="1"/>
          <speciesReference species="M_h2o_c" stoichiometry="1"/>
        </listOfProducts>
        <kineticLaw>
          <math xmlns="http://www.w3.org/1998/Math/MathML">
            <ci> FLUX_VALUE </ci>
          </math>
          <listOfParameters>
            <parameter id="LOWER_BOUND" value="-100"
units="mmol_per_gDW_per_hr"/>
            <parameter id="UPPER_BOUND" value="100"
units="mmol_per_gDW_per_hr"/>
            <parameter id="FLUX_VALUE" value="0"
units="mmol_per_gDW_per_hr"/>
            <parameter id="OBJECTIVE_COEFFICIENT" value="0"
units="mmol_per_gDW_per_hr"/>
          </listOfParameters>
        </kineticLaw>
      </reaction>
      <reaction id="R_pyruvate_carboxylase" name="1 pyr[c] + 1 atp[c] + 1
co2[c] = 1 adp[c] + 1 oaa[c]" reversible="1">
        <notes>
          <html:p>GENE_ASSOCIATION:  DVU1834</html:p>
          <html:p>SUBSYSTEM:  TCA</html:p>
          <html:p>note: </html:p>
          <html:p>Equation: 1 pyr[c] + 1 atp[c] + 1 co2[c] = 1 adp[c] + 1
oaa[c]</html:p>
        </notes>
        <listOfReactants>
          <speciesReference species="M_pyr_c" stoichiometry="1"/>
          <speciesReference species="M_atp_c" stoichiometry="1"/>
          <speciesReference species="M_co2_c" stoichiometry="1"/>
        </listOfReactants>
        <listOfProducts>
          <speciesReference species="M_adp_c" stoichiometry="1"/>
          <speciesReference species="M_oaa_c" stoichiometry="1"/>
        </listOfProducts>
        <kineticLaw>
          <math xmlns="http://www.w3.org/1998/Math/MathML">
            <ci> FLUX_VALUE </ci>
          </math>
          <listOfParameters>
            <parameter id="LOWER_BOUND" value="-100"
units="mmol_per_gDW_per_hr"/>
            <parameter id="UPPER_BOUND" value="100"
units="mmol_per_gDW_per_hr"/>
            <parameter id="FLUX_VALUE" value="0"
units="mmol_per_gDW_per_hr"/>
            <parameter id="OBJECTIVE_COEFFICIENT" value="0"
units="mmol_per_gDW_per_hr"/>
          </listOfParameters>
        </kineticLaw>
      </reaction>
      <reaction id="R_malate_dehydrogenase" name="1 pyr[c] + 1 co2[c] + 1
nadh[c] = 1 mal[c] + 1 nad[c]" reversible="1">
        <notes>
          <html:p>GENE_ASSOCIATION:  DVU0414</html:p>
          <html:p>SUBSYSTEM:  TCA</html:p>
          <html:p>note: </html:p>
```

```
          <html:p>Equation: 1 pyr[c] + 1 co2[c] + 1 nadh[c] = 1 mal[c] + 1
nad[c]</html:p>
        </notes>
        <listOfReactants>
          <speciesReference species="M_pyr_c" stoichiometry="1"/>
          <speciesReference species="M_co2_c" stoichiometry="1"/>
          <speciesReference species="M_nadh_c" stoichiometry="1"/>
        </listOfReactants>
        <listOfProducts>
          <speciesReference species="M_mal_c" stoichiometry="1"/>
          <speciesReference species="M_nad_c" stoichiometry="1"/>
        </listOfProducts>
        <kineticLaw>
          <math xmlns="http://www.w3.org/1998/Math/MathML">
            <ci> FLUX_VALUE </ci>
          </math>
          <listOfParameters>
            <parameter id="LOWER_BOUND" value="-100"
units="mmol_per_gDW_per_hr"/>
            <parameter id="UPPER_BOUND" value="100"
units="mmol_per_gDW_per_hr"/>
            <parameter id="FLUX_VALUE" value="0"
units="mmol_per_gDW_per_hr"/>
            <parameter id="OBJECTIVE_COEFFICIENT" value="0"
units="mmol_per_gDW_per_hr"/>
          </listOfParameters>
        </kineticLaw>
      </reaction>
      <reaction id="R_hydrogen_transferase" name="1 h2[c] = 1 h2[e]"
reversible="1">
        <notes>
          <html:p>GENE_ASSOCIATION:  </html:p>
          <html:p>SUBSYSTEM:  transport</html:p>
          <html:p>note: </html:p>
          <html:p>Equation: 1 h2[c] = 1 h2[e]</html:p>
        </notes>
        <listOfReactants>
          <speciesReference species="M_h2_c" stoichiometry="1"/>
        </listOfReactants>
        <listOfProducts>
          <speciesReference species="M_h2_e" stoichiometry="1"/>
        </listOfProducts>
        <kineticLaw>
          <math xmlns="http://www.w3.org/1998/Math/MathML">
            <ci> FLUX_VALUE </ci>
          </math>
          <listOfParameters>
            <parameter id="LOWER_BOUND" value="-100"
units="mmol_per_gDW_per_hr"/>
            <parameter id="UPPER_BOUND" value="100"
units="mmol_per_gDW_per_hr"/>
            <parameter id="FLUX_VALUE" value="0"
units="mmol_per_gDW_per_hr"/>
            <parameter id="OBJECTIVE_COEFFICIENT" value="0"
units="mmol_per_gDW_per_hr"/>
          </listOfParameters>
        </kineticLaw>
      </reaction>
      <reaction id="R_lactate_transferase" name="1 lac[c] = 1 lac[e]"
reversible="1">
        <notes>
```

```
          <html:p>GENE_ASSOCIATION:  </html:p>
          <html:p>SUBSYSTEM:  transport</html:p>
          <html:p>note: </html:p>
          <html:p>Equation: 1 lac[c] = 1 lac[e]</html:p>
        </notes>
        <listOfReactants>
          <speciesReference species="M_lac_c" stoichiometry="1"/>
        </listOfReactants>
        <listOfProducts>
          <speciesReference species="M_lac_e" stoichiometry="1"/>
        </listOfProducts>
        <kineticLaw>
          <math xmlns="http://www.w3.org/1998/Math/MathML">
            <ci> FLUX_VALUE </ci>
          </math>
          <listOfParameters>
            <parameter id="LOWER_BOUND" value="-100"
units="mmol_per_gDW_per_hr"/>
            <parameter id="UPPER_BOUND" value="100"
units="mmol_per_gDW_per_hr"/>
            <parameter id="FLUX_VALUE" value="0"
units="mmol_per_gDW_per_hr"/>
            <parameter id="OBJECTIVE_COEFFICIENT" value="0"
units="mmol_per_gDW_per_hr"/>
          </listOfParameters>
        </kineticLaw>
      </reaction>
      <reaction id="R_acetate_transferase" name="1 ac[c] = 1 ac[e]"
reversible="1">
        <notes>
          <html:p>GENE_ASSOCIATION:  </html:p>
          <html:p>SUBSYSTEM:  transport</html:p>
          <html:p>note: </html:p>
          <html:p>Equation: 1 ac[c] = 1 ac[e]</html:p>
        </notes>
        <listOfReactants>
          <speciesReference species="M_ac_c" stoichiometry="1"/>
        </listOfReactants>
        <listOfProducts>
          <speciesReference species="M_ac_e" stoichiometry="1"/>
        </listOfProducts>
        <kineticLaw>
          <math xmlns="http://www.w3.org/1998/Math/MathML">
            <ci> FLUX_VALUE </ci>
          </math>
          <listOfParameters>
            <parameter id="LOWER_BOUND" value="-100"
units="mmol_per_gDW_per_hr"/>
            <parameter id="UPPER_BOUND" value="100"
units="mmol_per_gDW_per_hr"/>
            <parameter id="FLUX_VALUE" value="0"
units="mmol_per_gDW_per_hr"/>
            <parameter id="OBJECTIVE_COEFFICIENT" value="0"
units="mmol_per_gDW_per_hr"/>
          </listOfParameters>
        </kineticLaw>
      </reaction>
      <reaction id="R_sulfate_transferase" name="1 sulfate[c] = 1
sulfate[e]" reversible="1">
        <notes>
          <html:p>GENE_ASSOCIATION:  </html:p>
```

```
          <html:p>SUBSYSTEM:  transport</html:p>
          <html:p>note: </html:p>
          <html:p>Equation: 1 sulfate[c] = 1 sulfate[e]</html:p>
        </notes>
        <listOfReactants>
          <speciesReference species="M_sulfate_c" stoichiometry="1"/>
        </listOfReactants>
        <listOfProducts>
          <speciesReference species="M_sulfate_e" stoichiometry="1"/>
        </listOfProducts>
        <kineticLaw>
          <math xmlns="http://www.w3.org/1998/Math/MathML">
            <ci> FLUX_VALUE </ci>
          </math>
          <listOfParameters>
            <parameter id="LOWER_BOUND" value="-100"
units="mmol_per_gDW_per_hr"/>
            <parameter id="UPPER_BOUND" value="100"
units="mmol_per_gDW_per_hr"/>
            <parameter id="FLUX_VALUE" value="0"
units="mmol_per_gDW_per_hr"/>
            <parameter id="OBJECTIVE_COEFFICIENT" value="0"
units="mmol_per_gDW_per_hr"/>
          </listOfParameters>
        </kineticLaw>
      </reaction>
      <reaction id="R_ethanol_transferase" name="1 etoh[c] = 1 etoh[e]"
reversible="1">
        <notes>
          <html:p>GENE_ASSOCIATION:  </html:p>
          <html:p>SUBSYSTEM:  transport</html:p>
          <html:p>note: </html:p>
          <html:p>Equation: 1 etoh[c] = 1 etoh[e]</html:p>
        </notes>
        <listOfReactants>
          <speciesReference species="M_etoh_c" stoichiometry="1"/>
        </listOfReactants>
        <listOfProducts>
          <speciesReference species="M_etoh_e" stoichiometry="1"/>
        </listOfProducts>
        <kineticLaw>
          <math xmlns="http://www.w3.org/1998/Math/MathML">
            <ci> FLUX_VALUE </ci>
          </math>
          <listOfParameters>
            <parameter id="LOWER_BOUND" value="-100"
units="mmol_per_gDW_per_hr"/>
            <parameter id="UPPER_BOUND" value="100"
units="mmol_per_gDW_per_hr"/>
            <parameter id="FLUX_VALUE" value="0"
units="mmol_per_gDW_per_hr"/>
            <parameter id="OBJECTIVE_COEFFICIENT" value="0"
units="mmol_per_gDW_per_hr"/>
          </listOfParameters>
        </kineticLaw>
      </reaction>
      <reaction id="R_phosphate_transferase" name="1 pi[c] = 1 pi[e]"
reversible="1">
        <notes>
          <html:p>GENE_ASSOCIATION:  </html:p>
          <html:p>SUBSYSTEM:  transport</html:p>
```

```
          <html:p>note: </html:p>
          <html:p>Equation: 1 pi[c] = 1 pi[e]</html:p>
        </notes>
        <listOfReactants>
          <speciesReference species="M_pi_c" stoichiometry="1"/>
        </listOfReactants>
        <listOfProducts>
          <speciesReference species="M_pi_e" stoichiometry="1"/>
        </listOfProducts>
        <kineticLaw>
          <math xmlns="http://www.w3.org/1998/Math/MathML">
            <ci> FLUX_VALUE </ci>
          </math>
          <listOfParameters>
            <parameter id="LOWER_BOUND" value="-100"
units="mmol_per_gDW_per_hr"/>
            <parameter id="UPPER_BOUND" value="100"
units="mmol_per_gDW_per_hr"/>
            <parameter id="FLUX_VALUE" value="0"
units="mmol_per_gDW_per_hr"/>
            <parameter id="OBJECTIVE_COEFFICIENT" value="0"
units="mmol_per_gDW_per_hr"/>
          </listOfParameters>
        </kineticLaw>
      </reaction>
      <reaction id="R_hydrogen_sulfide_transferase" name="1 h2s[c] = 1
h2s[e]" reversible="1">
        <notes>
          <html:p>GENE_ASSOCIATION:  </html:p>
          <html:p>SUBSYSTEM:  transport</html:p>
          <html:p>note: </html:p>
          <html:p>Equation: 1 h2s[c] = 1 h2s[e]</html:p>
        </notes>
        <listOfReactants>
          <speciesReference species="M_h2s_c" stoichiometry="1"/>
        </listOfReactants>
        <listOfProducts>
          <speciesReference species="M_h2s_e" stoichiometry="1"/>
        </listOfProducts>
        <kineticLaw>
          <math xmlns="http://www.w3.org/1998/Math/MathML">
            <ci> FLUX_VALUE </ci>
          </math>
          <listOfParameters>
            <parameter id="LOWER_BOUND" value="-100"
units="mmol_per_gDW_per_hr"/>
            <parameter id="UPPER_BOUND" value="100"
units="mmol_per_gDW_per_hr"/>
            <parameter id="FLUX_VALUE" value="0"
units="mmol_per_gDW_per_hr"/>
            <parameter id="OBJECTIVE_COEFFICIENT" value="0"
units="mmol_per_gDW_per_hr"/>
          </listOfParameters>
        </kineticLaw>
      </reaction>
      <reaction id="R_formate_transferase" name="1 form[c] = 1 form[e]"
reversible="1">
        <notes>
          <html:p>GENE_ASSOCIATION:  </html:p>
          <html:p>SUBSYSTEM:  transport</html:p>
          <html:p>note: </html:p>
```

```
          <html:p>Equation: 1 form[c] = 1 form[e]</html:p>
        </notes>
        <listOfReactants>
          <speciesReference species="M_form_c" stoichiometry="1"/>
        </listOfReactants>
        <listOfProducts>
          <speciesReference species="M_form_e" stoichiometry="1"/>
        </listOfProducts>
        <kineticLaw>
          <math xmlns="http://www.w3.org/1998/Math/MathML">
            <ci> FLUX_VALUE </ci>
          </math>
          <listOfParameters>
            <parameter id="LOWER_BOUND" value="-100"
units="mmol_per_gDW_per_hr"/>
            <parameter id="UPPER_BOUND" value="100"
units="mmol_per_gDW_per_hr"/>
            <parameter id="FLUX_VALUE" value="0"
units="mmol_per_gDW_per_hr"/>
            <parameter id="OBJECTIVE_COEFFICIENT" value="0"
units="mmol_per_gDW_per_hr"/>
          </listOfParameters>
        </kineticLaw>
      </reaction>
      <reaction id="R_Co2_transferase" name="1 co2[c] = 1 co2[e]"
reversible="1">
        <notes>
          <html:p>GENE_ASSOCIATION:  </html:p>
          <html:p>SUBSYSTEM:  transport</html:p>
          <html:p>note: </html:p>
          <html:p>Equation: 1 co2[c] = 1 co2[e]</html:p>
        </notes>
        <listOfReactants>
          <speciesReference species="M_co2_c" stoichiometry="1"/>
        </listOfReactants>
        <listOfProducts>
          <speciesReference species="M_co2_e" stoichiometry="1"/>
        </listOfProducts>
        <kineticLaw>
          <math xmlns="http://www.w3.org/1998/Math/MathML">
            <ci> FLUX_VALUE </ci>
          </math>
          <listOfParameters>
            <parameter id="LOWER_BOUND" value="-100"
units="mmol_per_gDW_per_hr"/>
            <parameter id="UPPER_BOUND" value="100"
units="mmol_per_gDW_per_hr"/>
            <parameter id="FLUX_VALUE" value="0"
units="mmol_per_gDW_per_hr"/>
            <parameter id="OBJECTIVE_COEFFICIENT" value="0"
units="mmol_per_gDW_per_hr"/>
          </listOfParameters>
        </kineticLaw>
      </reaction>
      <reaction id="R_succinate_transferase" name="1 succ[c] = 1 succ[e]"
reversible="1">
        <notes>
          <html:p>GENE_ASSOCIATION:  </html:p>
          <html:p>SUBSYSTEM:  transport</html:p>
          <html:p>note: </html:p>
          <html:p>Equation: 1 succ[c] = 1 succ[e]</html:p>
```

```
        </notes>
        <listOfReactants>
          <speciesReference species="M_succ_c" stoichiometry="1"/>
        </listOfReactants>
        <listOfProducts>
          <speciesReference species="M_succ_e" stoichiometry="1"/>
        </listOfProducts>
        <kineticLaw>
          <math xmlns="http://www.w3.org/1998/Math/MathML">
            <ci> FLUX_VALUE </ci>
          </math>
          <listOfParameters>
            <parameter id="LOWER_BOUND" value="-100"
units="mmol_per_gDW_per_hr"/>
            <parameter id="UPPER_BOUND" value="100"
units="mmol_per_gDW_per_hr"/>
            <parameter id="FLUX_VALUE" value="0"
units="mmol_per_gDW_per_hr"/>
            <parameter id="OBJECTIVE_COEFFICIENT" value="0"
units="mmol_per_gDW_per_hr"/>
          </listOfParameters>
        </kineticLaw>
      </reaction>
      <reaction id="R_Water_diffuse" name="1 h2o[c] = 1 h2o[e]"
reversible="1">
        <notes>
          <html:p>GENE_ASSOCIATION:  </html:p>
          <html:p>SUBSYSTEM:  transport</html:p>
          <html:p>note: </html:p>
          <html:p>Equation: 1 h2o[c] = 1 h2o[e]</html:p>
        </notes>
        <listOfReactants>
          <speciesReference species="M_h2o_c" stoichiometry="1"/>
        </listOfReactants>
        <listOfProducts>
          <speciesReference species="M_h2o_e" stoichiometry="1"/>
        </listOfProducts>
        <kineticLaw>
          <math xmlns="http://www.w3.org/1998/Math/MathML">
            <ci> FLUX_VALUE </ci>
          </math>
          <listOfParameters>
            <parameter id="LOWER_BOUND" value="-100"
units="mmol_per_gDW_per_hr"/>
            <parameter id="UPPER_BOUND" value="100"
units="mmol_per_gDW_per_hr"/>
            <parameter id="FLUX_VALUE" value="0"
units="mmol_per_gDW_per_hr"/>
            <parameter id="OBJECTIVE_COEFFICIENT" value="0"
units="mmol_per_gDW_per_hr"/>
          </listOfParameters>
        </kineticLaw>
      </reaction>
      <reaction id="R_biomasse_transferase" name="1 biomasse[c] = 1
biomasse[e]" reversible="1">
        <notes>
          <html:p>GENE_ASSOCIATION:  </html:p>
          <html:p>SUBSYSTEM:  transport</html:p>
          <html:p>note: </html:p>
          <html:p>Equation: 1 biomasse[c] = 1 biomasse[e]</html:p>
        </notes>
```

```
        <listOfReactants>
          <speciesReference species="M_biomasse_c" stoichiometry="1"/>
        </listOfReactants>
        <listOfProducts>
          <speciesReference species="M_biomasse_e" stoichiometry="1"/>
        </listOfProducts>
        <kineticLaw>
          <math xmlns="http://www.w3.org/1998/Math/MathML">
            <ci> FLUX_VALUE </ci>
          </math>
          <listOfParameters>
            <parameter id="LOWER_BOUND" value="-100"
units="mmol_per_gDW_per_hr"/>
            <parameter id="UPPER_BOUND" value="100"
units="mmol_per_gDW_per_hr"/>
            <parameter id="FLUX_VALUE" value="0"
units="mmol_per_gDW_per_hr"/>
            <parameter id="OBJECTIVE_COEFFICIENT" value="0"
units="mmol_per_gDW_per_hr"/>
          </listOfParameters>
        </kineticLaw>
      </reaction>
      <reaction id="R_formiate_Cacetyltransferase" name="1 CoA[e] +
pyruvate[e] = 1 acetyl_CoA[e] + 1 formiate[e]" reversible="1">
        <notes>
          <html:p>GENE_ASSOCIATION:  </html:p>
          <html:p>SUBSYSTEM:  Wood</html:p>
          <html:p>note: </html:p>
          <html:p>Equation: 1 CoA[e] + pyruvate = 1 acetyl_CoA[e] + 1
formiate[e]</html:p>
        </notes>
        <listOfReactants>
          <speciesReference species="M_coa_c" stoichiometry="1"/>
          <speciesReference species="M_pyr_c" stoichiometry="1"/>
        </listOfReactants>
        <listOfProducts>
          <speciesReference species="M_accoa_c" stoichiometry="1"/>
          <speciesReference species="M_form_c" stoichiometry="1"/>
        </listOfProducts>
        <kineticLaw>
          <math xmlns="http://www.w3.org/1998/Math/MathML">
            <ci> FLUX_VALUE </ci>
          </math>
          <listOfParameters>
            <parameter id="LOWER_BOUND" value="-100"
units="mmol_per_gDW_per_hr"/>
            <parameter id="UPPER_BOUND" value="100"
units="mmol_per_gDW_per_hr"/>
            <parameter id="FLUX_VALUE" value="0"
units="mmol_per_gDW_per_hr"/>
            <parameter id="OBJECTIVE_COEFFICIENT" value="0"
units="mmol_per_gDW_per_hr"/>
          </listOfParameters>
        </kineticLaw>
      </reaction>
      <reaction id="R_formate_dehydrogenase" name="1 form[c] + 1 nad[c] -->
1 co2[c] + 1 nadh[c]" reversible="0">
        <notes>
          <html:p>GENE_ASSOCIATION:  DVU0587</html:p>
          <html:p>SUBSYSTEM:  Wood</html:p>
          <html:p>note: </html:p>
```

```
          <html:p>Equation: 1 form[c] + 1 nad[c] --> 1 co2[c] + 1
nadh[c]</html:p>
        </notes>
        <listOfReactants>
          <speciesReference species="M_form_c" stoichiometry="1"/>
          <speciesReference species="M_nad_c" stoichiometry="1"/>
        </listOfReactants>
        <listOfProducts>
          <speciesReference species="M_co2_c" stoichiometry="1"/>
          <speciesReference species="M_nadh_c" stoichiometry="1"/>
        </listOfProducts>
        <kineticLaw>
          <math xmlns="http://www.w3.org/1998/Math/MathML">
            <ci> FLUX_VALUE </ci>
          </math>
          <listOfParameters>
            <parameter id="LOWER_BOUND" value="0"
units="mmol_per_gDW_per_hr"/>
            <parameter id="UPPER_BOUND" value="100"
units="mmol_per_gDW_per_hr"/>
            <parameter id="FLUX_VALUE" value="0"
units="mmol_per_gDW_per_hr"/>
            <parameter id="OBJECTIVE_COEFFICIENT" value="0"
units="mmol_per_gDW_per_hr"/>
          </listOfParameters>
        </kineticLaw>
      </reaction>
      <reaction id="R_hydrolyse_biphosphate" name="1 ppi[c] + 1 h2o[c] -->
2 pi[c]" reversible="0">
        <notes>
          <html:p>GENE_ASSOCIATION:  </html:p>
          <html:p>SUBSYSTEM:  phosphate</html:p>
          <html:p>note: </html:p>
          <html:p>Equation: 1 ppi[c] + 1 h2o[c] --> 2 pi[c]</html:p>
        </notes>
        <listOfReactants>
          <speciesReference species="M_ppi_c" stoichiometry="1"/>
          <speciesReference species="M_h2o_c" stoichiometry="1"/>
        </listOfReactants>
        <listOfProducts>
          <speciesReference species="M_pi_c" stoichiometry="2"/>
        </listOfProducts>
        <kineticLaw>
          <math xmlns="http://www.w3.org/1998/Math/MathML">
            <ci> FLUX_VALUE </ci>
          </math>
          <listOfParameters>
            <parameter id="LOWER_BOUND" value="0"
units="mmol_per_gDW_per_hr"/>
            <parameter id="UPPER_BOUND" value="100"
units="mmol_per_gDW_per_hr"/>
            <parameter id="FLUX_VALUE" value="0"
units="mmol_per_gDW_per_hr"/>
            <parameter id="OBJECTIVE_COEFFICIENT" value="0"
units="mmol_per_gDW_per_hr"/>
          </listOfParameters>
        </kineticLaw>
      </reaction>
      <reaction id="R_adp_transferase" name="1 adp[c] = 1 adp[e]"
reversible="1">
        <notes>
```

```
          <html:p>GENE_ASSOCIATION:  </html:p>
          <html:p>SUBSYSTEM:  transport</html:p>
          <html:p>note: </html:p>
          <html:p>Equation: 1 adp[c] = 1 adp[e]</html:p>
        </notes>
        <listOfReactants>
          <speciesReference species="M_adp_c" stoichiometry="1"/>
        </listOfReactants>
        <listOfProducts>
          <speciesReference species="M_adp_e" stoichiometry="1"/>
        </listOfProducts>
        <kineticLaw>
          <math xmlns="http://www.w3.org/1998/Math/MathML">
            <ci> FLUX_VALUE </ci>
          </math>
          <listOfParameters>
            <parameter id="LOWER_BOUND" value="-100"
units="mmol_per_gDW_per_hr"/>
            <parameter id="UPPER_BOUND" value="100"
units="mmol_per_gDW_per_hr"/>
            <parameter id="FLUX_VALUE" value="0"
units="mmol_per_gDW_per_hr"/>
            <parameter id="OBJECTIVE_COEFFICIENT" value="0"
units="mmol_per_gDW_per_hr"/>
          </listOfParameters>
        </kineticLaw>
      </reaction>
      <reaction id="R_adp_EX" name="1 adp[e] = " reversible="1">
        <notes>
          <html:p>GENE_ASSOCIATION:  </html:p>
          <html:p>SUBSYSTEM:  base</html:p>
          <html:p>note: </html:p>
          <html:p>Equation: 1 adp[e] =</html:p>
        </notes>
        <listOfReactants>
          <speciesReference species="M_adp_e" stoichiometry="1"/>
        </listOfReactants>
        <kineticLaw>
          <math xmlns="http://www.w3.org/1998/Math/MathML">
            <ci> FLUX_VALUE </ci>
          </math>
          <listOfParameters>
            <parameter id="LOWER_BOUND" value="-100"
units="mmol_per_gDW_per_hr"/>
            <parameter id="UPPER_BOUND" value="100"
units="mmol_per_gDW_per_hr"/>
            <parameter id="FLUX_VALUE" value="0"
units="mmol_per_gDW_per_hr"/>
            <parameter id="OBJECTIVE_COEFFICIENT" value="0"
units="mmol_per_gDW_per_hr"/>
          </listOfParameters>
        </kineticLaw>
      </reaction>
    </listOfReactions>
  </model>
</sbml>
```
