# Supplementary material for: Combining metabolic flux analysis with proteomics to shed light on the metabolic flexibility: the case of Desulfovibrio vulgaris Hildenborough
Source: Front Microbiol. 2024 Feb 23;15:1336360. doi: 10.3389/fmicb.2024.1336360 (PMC10920352; doi:10.3389/fmicb.2024.1336360)
Supplement: Supplementary file 3 [file Data_Sheet_3.docx]

Supplementary Material

# Supplementary Data

Supplementary Data S1: Overview of the reconstructed metabolic model iDvu71.

Supplementary Data S2: Whole proteome from the soluble and membrane fractions of *Dv*H grown under lactate/sulfate respiration.

# Supplementary Figures and Tables

## Supplementary Figures

**Supplementary Figure 1.** **Example of organic acids produced in the cell suspension during sulfate respiration using lactate as carbon and energy source.** 25 μL of of clarified cell supernatant at different time points (from dark green to light green) were applied to Hiplex H column at 50 ºC, using sulfuric acid as mobile phase (0.6 mL.min ^-1^) as described in the method section. Separated compounds were detected using a refractive index detector. Over the different organic acids analysed, only lactate (16.2 min retention time) was produced. This is concomitant with lactate utilization (13.8 min retention time). Among the other peaks detected, one unidentified compound (retention time 9.5 min) is also utilized over growth. However, attempts to identify these two peaks gave no clear results and its nature remain to be determined.


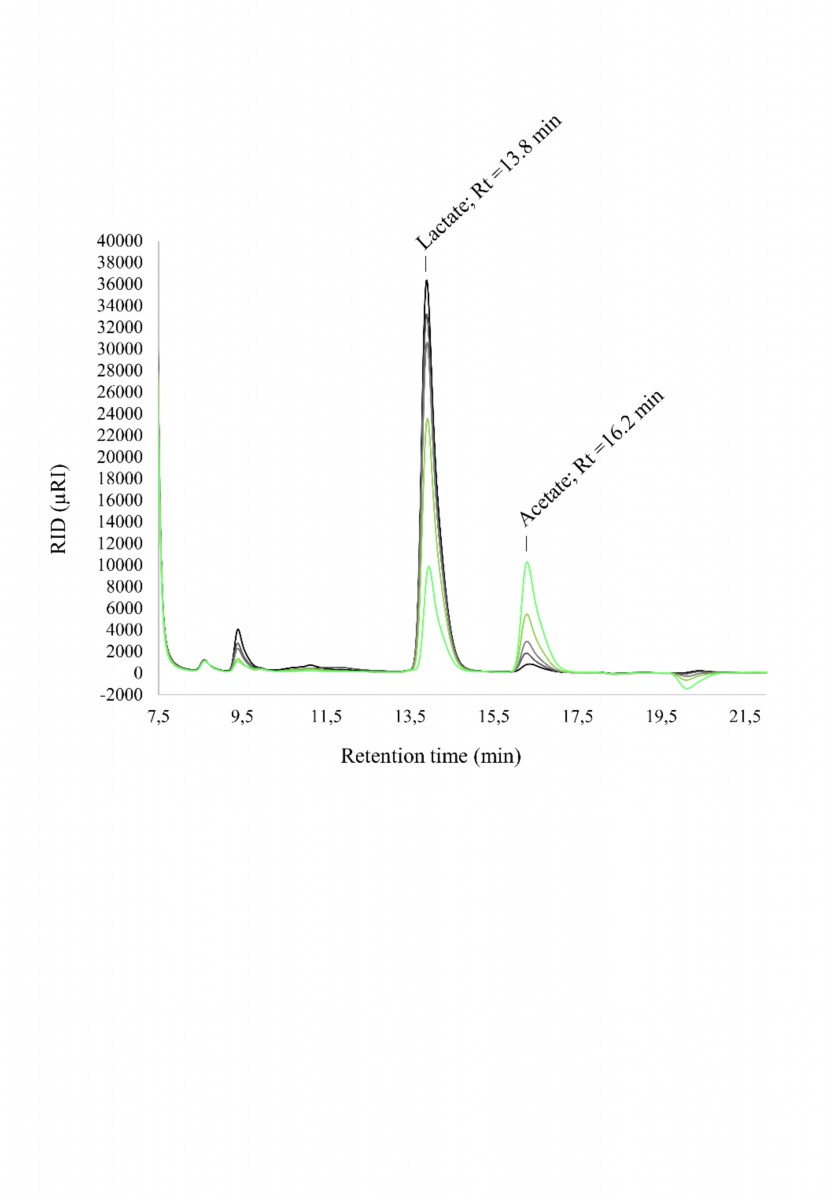


## Supplementary Tables:

| **Enzymes** | **Subunit/**  **Protein** | **Accession** | **Locus tag** | **MW** | **PSM 1** | **PSM 2** | **PSM 3** |
| --- | --- | --- | --- | --- | --- | --- | --- |
| **Lactate utilization** |  |  |  |  |  |  |  |
| Pyruvate:ferredoxin oxidoreductase | | | | | | | |
| subunit A | PorA | Q72BR5 | DVU_1569 | 61.8 | 13 | 15 | 20 |
| subunit B | PorB | Q72BR4 | DVU_1570 | 30.8 | 10 | 5 | 7 |
| Pyruvate::ferredoxin oxidoreductase | PFOR | Q726T1 | DVU_3025 | 30.8 | 402 | 409 | 439 |
| L-lactate permease | Ltp | Q726T0 | DVU_3026 | 60.8 | 6 | 7 | 7 |
| D-lactate dehydrogenase  subunit A | D-LdII-A | Q726S9 | DVU_3027 | 49.3 | 85 | 97 | 114 |
| subunit B | D-LdII-B | Q726S8 | DVU_3028 | 45.9 | 32 | 36 | 40 |
| Phosphate acetyl transferase | Pta | Q726S7 | DVU_3029 | 76.8 | 121 | 140 | 164 |
| Acetate kinase | Ack | Q726S6 | DVU_3030 | 44.1 | 77 | 75 | 85 |
| Conserved hypothetical protein |  | Q726S5 | DVU_3031 | 39.0 | 12 | 75 | 5 |
| L-lactate dehydrogenase  subunit A | LldG | Q726S4 | DVU_3032 | 22.6 | 21 | 26 | 25 |
| L-lactate dehydrogenase subunit B | LldH | Q726S3 | DVU_3033 | 79.6 | 106 | 80 | 82 |
| Lactate dehydrogenase |  | P62051 | DVU_0600 | 32.2 | 2 | 1 | 1p |
| D-lactate dehydrogenase  subunit A (Dld-II family) |  | [Q72DV3](https://www.uniprot.org/uniprot/Q72DV3) | DVU_0826 | 47.4 | 2 | 3 | 2 |
| subunit B (Dld-II family) |  | [Q72DV2](https://www.uniprot.org/uniprot/Q72DV2) | DVU_0827 | 50.1 | 7 | 3 | 3 |
| D-lactate dehydrogenase  (Dld-II family) |  | Q72F25 | DVU_0390 | 48.2 | 25 | 13 | 11 |
| D-lactate dehydrogenase  (Dld-II family) |  | Q72FG1 | DVU_0253 | 103 | 88 | 74 | 88 |
| L-lactate dehydrogenase  (LldG family) |  | [Q72B57](https://www.uniprot.org/uniprot/Q72B57) | DVU_1781 | 23.9 | - | - | - |
| L-lactate dehydrogenase  (LldH family) |  | [Q72B56](https://www.uniprot.org/uniprot/Q72B56) | DVU_1782 | 52.6 | 4 | 2 | 2 |
| Lactate permeases, putative |  | Q72B55 | DVU_1783 | 27.4 | 3 | - | 1 |
|  |  | Q72A87 | DVU_2110 | 57.9 | 5 | 3 | 4 |
|  |  | Q729R4 | DVU_2285 | 53.0 | 5 | 8 | 6 |
|  |  | Q725Z0 | DVU_3284 | 57.7 | - | - | - |
| **Sulfate reduction** |  |  |  |  |  |  |  |
| Sulfate permeases |  | Q72FD5 | DVU_0279 | 60.6 | 9 | 7 | 8 |
|  |  | Q72G10 | DVU_0053 | 68.1 | 5 | 8 | 7 |
| Sulfate adenylyl transferase | Sat | Q72CI8 | DVU_1295 | 47.4 | 301 | 567 | 565 |
| Adenylyl sulfate reductase AprAB | | | | | | | |
| Subunit B | AprB | Q72DT3 | DVU_0846 | 18.5 | 47 | 32 | 42 |
| subunit A | AprA | Q72DT2 | DVU_0847 | 74.6 | 545 | 484 | 550 |
| Sulfite reductase, DsrABC complex | | | | | | | |
| Subunit A | DsrA | P45574 | DVU_0402 | 49.1 | 153 | 156 | 156 |
| Subunit B | DsrB | P45575 | DVU_0403 | 42.5 | 117 | 191 | 243 |
| Subunit D | DsvD | Q46582 | DVU_0404 | 8.8 | 7 | - | - |
| Subunit C | DsrC | P45573 | DVU_2776 | 11.9 | 14 | 17 | 29 |
| Quinone oxidoreductase QmoABC complex | | | | | | | |
| subunit A | QmoA | Q72DT1 | DVU_0848 | 44.6 | 82 | 62 | 76 |
| subunit B | QmoB | Q72DT0 | DVU_0849 | 82.5 | 211 | 116 | 156 |
| subunit C | QmoC | Q72DS9 | DVU_0850 | 42.6 | 46 | 26 | 42 |
| Hypothetical protein | QmoD | Q72DS8 | DVU_0851 | 25.8 | 63 | 29 | 48 |
| Dissimilatory sulfite reductase DsrMKJOP complex | | | | | | | |
| subunit P | DsrP | Q72CJ7 | DVU_1286 | 43.5 | - | - | 3 |
| subunit O | DsrO | Q72CJ6 | DVU_1287 | 29.0 | 28 | 22 | 28 |
| subunit J | DsrJ | Q72CJ5 | DVU_1288 | 14.5 | - | 3 | 4 |
| subunit K | DsrK | Q72CJ4 | DVU_1289 | 60.7 | 40 | 47 | 44 |
| subunit M | DsrM | Q72CJ3 | DVU_1290 | 38.1 | 30 | 21 | 27 |

| **Enzymes** | **Subunit/**  **Protein** | **Accession** | **Locus tag** | **MW** | **PSM 1** | **PSM 2** | **PSM 3** |
| --- | --- | --- | --- | --- | --- | --- | --- |
| **Hydrogen metabolism** |  |  |  |  |  |  |  |
| Membrane-bound hydrogenase Ech complex | | | | | | | |
| subunit A | EchA | Q72EY4 | DVU_0434 | 69.1 | 4 | 4 | 2 |
| subunit B | EchB | Q72EY5 | DVU_0433 | 31.2 | - | 1 | 1 |
| subunit C | EchC | Q72EY6 | DVU_0432 | 16.8 | - | - | - |
| subunit D | EchD | Q72EY7 | DVU_0431 | 14.6 | 2 | - | - |
| subunit E | EchE | Q72EY8 | DVU_0430 | 40.1 | 5 | 9 | 7 |
| subunit F | EchF | Q72EY9 | DVU_0429 | 14.8 | 2 | 2 | 2 |
| Membrane-bound hydrogenase Coo complex | | | | | | | |
| subunit M | CooM | Q729R3 | DVU_2286 | 131.0 | 12 | 9 | 12 |
| subunit K | CooK | Q729R2 | DVU_2287 | 33.7 | 7 | 8 | 9 |
| subunit L | CooL | Q729R1 | DVU_2288 | 15.7 | 3 | 1 | 1 |
| subunit X | CooX | Q729R0 | DVU_2289 | 22.7 | 6 | - | - |
| subunit U | CooU | Q729Q9 | DVU_2290 | 19.6 | 5 | - | - |
| subunit H | CooH | Q729Q8 | DVU_2291 | 40.7 | 25 | 26 | 27 |
| Hydrogenase nickel-insertion protein | HypA | Q729Q7 | DVU_2292 | 12.7 | - | - | - |
| subunit F | CooF | Q729Q6 | DVU_2293 | 19.0 | - | - | - |
| [FeFe]-hydrogenase, Hyd complex | | | | | | | |
| subunit L | HydL | P07598 | DVU_1769 | 45.9 | - | 2 | 4 |
| subunit S | HydS | P07603 | DVU_1770 | 13.6 | - | - | - |
| [NiFeSe]-hydrogenase, Hys complex | | | | | | | |
| subunit B | HysB | Q72AS4 | DVU_1917 | 33.9 | 15 | 25 | 25 |
| subunit A | HysA | Q72AS3 | DVU_1918 | 55.8 | 96 | 119 | 130 |
| [NiFe]-hydrogenase, Hyn1 complex | | | | | | | |
| subunit B | HynB1 | Q06173 | DVU_1921 | 34.2 | - | - | - |
| subunit A | HynA1 | Q72AS0 | DVU_1922 | 62.7 | 25 | 18 | 17 |
| [NiFe]-hydrogenase, Hyn2 complex | | | | | | | |
| subunit B | HynB2 | P61429 | DVU_2525 | 34.5 | - | - | - |
| subunit A | HynA2 | Q728S7 | DVU_2526 | 61.2 | - | - | - |
| **Formate metabolism** |  |  |  |  |  |  |  |
| Pyruvate formate lyase activating enzyme, putative |  | Q729S8 | DVU_2271 | 32.4 | - | - | - |
| Pyruvate formate lyase, putative |  | Q729S7 | DVU_2272 | 91.2 | 13 | 2 | 2 |
| Pyruvate formate lyase, putative |  | Q727N1 | DVU_2824 | 93.9 | 28 | 15 | 17 |
| Pyruvate formate lyase activating enzyme, putative |  | Q727N0 | DVU_2825 | 33.7 | - | - | 1 |
| Formate dehydrogenase FdhABC_3_ complex, | | | | | | | |
| cytochrome *c_3_* subunit | Fdhc_3_ | Q727P6 | DVU_2809 | 15.8 | - | - | - |
| Formate dehydrogenase formation protein | FdhE | Q727P5 | DVU_2810 | 29.6 | - | - | - |
| subunit B | FdhB | Q727P4 | DVU_2811 | 24.5 | 2 | 4 | 3 |
| subunit A | FdhA | Q727P3 | DVU_2812 | 113.3 | 52 | 44 | 40 |
| Formate dehydrogenase FdhAB complex |  |  |  |  |  |  |  |
| subunit α | Fdhα | Q72EJ1 | DVU_0587 | 111.2 | - | 3 | - |
| subunit β | Fdhβ | Q72EJ0 | DVU_0588 | 26.4 | - |  | - |
| Formate dehydrogenase FdhM complex |  |  |  |  |  |  |  |
| subunit B | CfdB | Q728X1 | DVU_2481 | 29.2 | 2 | 3 | 8 |
| subunit A | CfdA | Q728X0 | DVU_2482 | 110.8 | 48 | 30 | 40 |
| cytochrome *c* putative | CfdE | Q728W9 | DVU_2483 | 61.9 | 11 | 11 | 12 |
| cytochrome *c* putative | CfdD | Q728W8 | DVU_2484 | 47.7 | 7 | 7 | 7 |

| **Enzymes** | **Subunit/**  **Protein** | | **Accession** | | | **Locus tag** | | | | **MW** | | | **PSM 1** | | **PMS 2** | | **PSM 3** |
| --- | --- | --- | --- | --- | --- | --- | --- | --- | --- | --- | --- | --- | --- | --- | --- | --- | --- |
| **Transmembrane electron transport complexes** | | | | | |  | | | |  | | |  | |  | |  |
| Quinone reductase complex  QrcDCBA | | | | | | | | | | | | | | | | | |
| subunit D | QrcD | | Q72E86 | | | DVU_0692 | | | | 47.5 | | | 8 | | 11 | | 13 |
| subunit C | QrcC | | Q72E85 | | | DVU_0693 | | | | 29.0 | | | 16 | | 19 | | 12 |
| subunit B | QrcB | | Q72E84 | | | DVU_0694 | | | | 72.3 | | | 105 | | 131 | | 136 |
| subunit A | QrcA | | Q72E83 | | | DVU_0695 | | | | 7.4 | | | - | | - | | - |
| Transmembrane complex TmcABCD | | |  | | |  | | | |  | | |  | | - | |  |
| subunit A | TmcA | | Q72FF1 | | | DVU_0263 | | | | 13.5 | | | - | | - | |  |
| subunit B | TmcB | | Q72FF0 | | | DVU_0264 | | | | 49.6 | | | 46 | | 44 | | 44 |
| subunit C | TmcC | | Q72FE9 | | | DVU_0265 | | | | 24.0 | | | 8 | | 7 | | 22 |
| subunit D | TmcC | | Q72FE8 | | | DVU_0266 | | | | 44.9 | | | 19 | | 31 | | 39 |
| High molecular weight cytochrome complex Hmc | | | |  | | |  | | |  | | |  | |  | |  |
| protein 6 | HmcF | | P33393 | | | DVU_0531 | | | | 52.7 | | | 29 | | 7 | | 9 |
| protein 5 | HmcE | | P33392 | | | DVU_0532 | | | | 25.3 | | | - | | - | | 1 |
| protein 4 | HmcD | | P33391 | | | DVU_0533 | | | | 5.8 | | | - | | - | | - |
| protein 3 | HmcC | | P33390 | | | DVU_0534 | | | | 43.1 | | | 2 | | 1 | | 1 |
| protein 2 | HmcB | | P33389 | | | DVU_0535 | | | | 40.0 | | | 18 | | 10 | | 10 |
| protein 1 | HmcA | | P24092 | | | DVU_0536 | | | | 58.9 | | | 12 | | 9 | | 12 |
| Rnf complex | |  | | |  | | |  |  | | | | | | | | |
| cytochrome *c* | DhcA | | Q727R4 | | | DVU_2791 | | | | 27.8 | | | - | | - | | - |
| subunit C | RnfC | | Q727R3 | | | DVU_2792 | | | | 43.5 | | | 26 | | 30 | | 28 |
| subunit D | RnfD | | Q727R2 | | | DVU_2793 | | | | 33.7 | | | - | | - | | - |
| subunit G | RndG | | Q727R1 | | | DVU_2794 | | | | 20.1 | | | 8 | | 8 | | 8 |
| subunit E | RnfE | | Q727R0 | | | DVU_2795 | | | | 23.8 | | | 5 | | 6 | | 5 |
| subunit A | RnfA | | Q727Q9 | | | DVU_2796 | | | | 20.8 | | | - | | - | | - |
| subunit B | RnfB | | Q727Q8 | | | DVU_2797 | | | | 30.9 | | | 4 | | 11 | | 6 |
| Membrane associated lipoprotein | ApbE | | Q727Q7 | | | DVU_2798 | | | | 35.0 | | | - | | - | | - |
| **Ethanol metabolism** |  | |  | | |  | | | |  | | |  | |  | |  |
| Aldehyde oxidoreductase | Aor | | Q72BS5 | | | DVU_1559 | | | | 97.4 | | | 66 | | 49 | | 67 |
| Iron-containing alcohol dehydrogenases | |  | | | | | | | | | | | | | | | |
|  | Adh1 | | Q729E6 | | | DVU_2405 | | | | | 41.7 | 111 | | 128 | | 139 | |
|  |  | | Q729Z6 | | | DVU_2201 | | | | 41.9 | | | 37 | | 39 | | 43 |
|  |  | | Q72F61 | | | DVU_0353 | | | | 39.4 | | | 24 | | 39 | | 35 |
|  |  | | Q727H0 | | | DVU_2885 | | | | 42.0 | | | 7 | | 8 | | 8 |
|  |  | | Q727F0 | | | DVU_2905 | | | | 41.0 | | | 3 | | 3 | | 3 |
|  | Adh2 | | Q729F5 | | | DVU_2396 | | | | 41.0 | | | 2 | | 1 | | - |
|  |  | | Q728Q8 | | | DVU_2545 | | | | 40.0 | | | - | | 5 | | 3 |
| NADH oxidoreductase complex Flx | |  | | | | | | | | | | | | | | | |
| subunit A | FlxA | | Q729F2 | | | DVU_2399 | | | | 31.0 | | | 24 | | 17 | | 17 |
| subunit B | FlxB | | Q729F1 | | | DVU_2400 | | | | 39.4 | | | 20 | | 4 | | 7 |
| subunit CD | FlxCD | | Q729F0 | | | DVU_2401 | | | | 54.1 | | | 46 | | 22 | | 27 |
| Hetero disulfide reductase complex Hdr | |  | | | | | | | | | | | | | | | |
| Subunit A | HdrA | | Q729E9 | | | DVU_2402 | | | | 70.9 | | | 52 | | 16 | | 23 |
| Subunit B | HdrB | | Q729E8 | | | DVU_2403 | | | | 35.1 | | | 17 | | 7 | | 10 |
| Subunit C | HdrC | | Q729E7 | | | DVU_2404 | | | | 21.0 | | | 6 | | 2 | | 2 |

| **Enzymes** | **Subunit/**  **Protein** | | **Accession** | **Locus tag** | **MW** | | **PSM 1** | **PSM 2** | **PSM 3** |
| --- | --- | --- | --- | --- | --- | --- | --- | --- | --- |
| **Gluconeogenesis** | | | |  |  | |  |  |  |
| Pyruvate kinase | Pk | | Q728T8 | DVU_2514 | 50.4 | | 31 | 18 | 16 |
| Phosphoenolpyruvate synthase | PpsA | | Q72B07 | DVU_1833 | 132.6 | | 184 | 164 | 186 |
| Pyruvate carboxylase | Pc | | Q72B06 | DVU_1834 | 136.3 | | 111 | 62 | 66 |
| 2,3 bisphosphoglycerate phosphoglycerate mutase | PGM | | Q72BL6 | DVU_1619 | 54.1 | | 8 | 6 | 4 |
| Phosphoglycerate kinase | PGAM | | P62412 | DVU_2529 | 414 | | 41 | 38 | 42 |
| Glyceraldehyde 3 phosphate dehydrogenase | |  | | | |  |  |  |  |
|  | GAPDH | | Q72EL3 | DVU_0565 | 37.0 | | 28 | 40 | 35 |
|  | GAPDH | | Q72A53 | DVU_2144 | 35.6 | | 27 | 32 | 30 |
| Fructose bi-phosphate aldolase | FBP | | Q72A54 | DVU_2143 | 33.5 | | 22 | 25 | 26 |
| 6-phosphofructokinase | PFK | | Q72AD6 | DVU_2061 | 48.1 | | 20 | 18 | 19 |
| Glucose 6-phosphate isomerase | GPI | | Q725H4 | DVU_3222 | 48.7 | | 18 | 24 | 19 |
| **TCA cycle** |  | |  |  |  | |  |  |  |
| Aconitate hydratase | CitB | | Q72D65 | DVU_1064 | 67.5 | | 49 | 28 | 30 |
| Isocitrate dehydrogenase | CitC | | Q72EU1 | DVU_0477 | 41.5 | | 24 | 15 | 19 |
| Fumarate reductase (Fdr)  subunit C | FdrC | | Q726D0 | DVU_3261 | 23.7 | | - | - | 2 |
| subunit A | FdrA | | Q726C9 | DVU_3262 | 65.9 | | 19 | 17 | 21 |
| subunit B | FdrB | | Q726C8 | DVU_3263 | 27.8 | | 5 | 6 | 7 |
| Fumarate hydratase | FH | | Q726A9 | DVU_3264 | 29.5 | | 2 | 2 | 3 |
| Tartrate dehydratase |  | | Q726A8 | DVU_3265 | 19.4 | | - | 2 | 1 |

**Supplementary Table S1.** Protein abundance (indicated by spectral counting as PSM) for all replicates. Proteins were identified from the soluble and membrane fractions of *Dv*H grown to exponential phase under anaerobic conditions in SKY medium as described in the Material and Methods section. For each replicate, 1669, 1507 and 1512 proteins were identified, respectively. Protein abundance (as PSM) for all replicates were reported in this table. Accession: accession number in the UniProt database. MW: theoretical molecular weight in kDa. PSM: peptide spectrum match number (given by the algorithm and corresponding to the total number of identified peptide sequences for the protein, including those redundantly identified).
